# Rural‒urban disparities in household catastrophic health expenditure in Bangladesh: a multivariate decomposition analysis

**DOI:** 10.1186/s12939-024-02125-3

**Published:** 2024-02-27

**Authors:** Taslima Rahman, Dominic Gasbarro, Khorshed Alam, Khurshid Alam

**Affiliations:** 1 Murdoch Business School, Murdoch University, Perth, WA 6150 Australia; 2 Institute of Health Economics, University of Dhaka, Dhaka, 1000 Bangladesh; 3 School of Business, University of Southern Queensland, Toowoomba, QLD 4350 Australia

**Keywords:** Health expenditures, Financial hardship, Universal health care, Sustainable development goals, Rural health services, Healthcare disparities, Bangladesh, Low and middle income countries

## Abstract

**Background:**

Rural‒urban disparity in catastrophic healthcare expenditure (CHE) is a well-documented challenge in low- and middle-income countries, including Bangladesh, limiting financial protection and hindering the achievement of the Universal Health Coverage target of the United Nations Sustainable Development Goals. However, the factors driving this divide remain poorly understood. Therefore, this study aims to identify the key determinants of the rural‒urban disparity in CHE incidence in Bangladesh and their changes over time.

**Methods:**

We used nationally representative data from the latest three rounds of the Bangladesh Household Income and Expenditure Survey (2005, 2010, and 2016). CHE incidence among households seeking healthcare was measured using the normative food, housing, and utilities method. To quantify covariate contributions to the rural‒urban CHE gap, we employed the Oaxaca-Blinder multivariate decomposition approach, adapted by Powers et al*.* for nonlinear response models.

**Results:**

CHE incidence among rural households increased persistently during the study period (2005: 24.85%, 2010: 25.74%, 2016: 27.91%) along with a significant (*p*-value ≤ 0.01) rural‒urban gap (2005: 9.74%-points, 2010: 13.94%-points, 2016: 12.90%-points). Despite declining over time, substantial proportions of CHE disparities (2005: 87.93%, 2010: 60.44%, 2016: 61.33%) are significantly (*p*-value ≤ 0.01) attributable to endowment differences between rural and urban households. The leading (three) covariate categories consistently contributing significantly (*p*-value ≤ 0.01) to the CHE gaps were composition disparities in the lowest consumption quintile (2005: 49.82%, 2010: 36.16%, 2016: 33.61%), highest consumption quintile (2005: 32.35%, 2010: 15.32%, 2016: 18.39%), and exclusive reliance on informal healthcare sources (2005: -36.46%, 2010: -10.17%, 2016: -12.58%). Distinctively, the presence of chronic illnesses in households emerged as a significant factor in 2016 (9.14%, *p*-value ≤ 0.01), superseding the contributions of composition differences in household heads with no education (4.40%, *p*-value ≤ 0.01) and secondary or higher education (7.44%, *p*-value ≤ 0.01), which were the fourth and fifth significant contributors in 2005 and 2010.

**Conclusions:**

Rural‒urban differences in household economic status, educational attainment of household heads, and healthcare sources were the key contributors to the rural‒urban CHE disparity between 2005 and 2016 in Bangladesh, with chronic illness emerging as a significant factor in the latest period. Closing the rural‒urban CHE gap necessitates strategies that carefully address rural‒urban variations in the characteristics identified above.

**Supplementary Information:**

The online version contains supplementary material available at 10.1186/s12939-024-02125-3.

## Introduction

Ensuring protection from the financial risks of seeking healthcare is a fundamental health system goal and, as outlined in the United Nations (UN) Sustainable Development Goals (SDGs), is a key component of Universal Health Coverage (UHC), the SDG target 3.8 [1, 2]. Achieving UHC requires a health system to ensure that everyone, regardless of their economic status, geographic location, or other characteristics, has equitable access to essential, high-quality healthcare without financial hardships [3]. While several indicators (such as catastrophic out-of-pocket (OOP) healthcare expenditure, impoverishment due to OOP expenses, adoption of coping strategies, and forgoing care for financial reasons) quantify the extent of financial hardships or the lack of financial protection among a population, the SDG framework, and consequently, most of the financial protection literature use the incidence of catastrophic healthcare expenditure (CHE) as the financial protection indicator [4–6].

OOP expenses at the point of service become catastrophic if they are disproportionately high relative to household resources or capacity-to-pay (CTP) for healthcare, compelling households to make undesirable trade-offs with other necessities (e.g., food) and ultimately reducing their living standards [7–9]. Several methods have evolved over the years to measure CHE, depending on the definition adopted to calculate household CTP [5]. Although the SDG target 3.8 pledges to reduce the proportion of people incurring CHE to zero by 2030, the global CHE trend is moving in the opposite direction, with the number of people incurring financial hardships remaining unacceptably high. According to recent estimates, nearly a billion (996 million) of the world's population incurred CHE in 2017, an increase of 56 million from 2015. Approximately 43% of the people incurring CHE in 2017 were from lower-middle-income countries (LwMICs), out of which 70% live in South Asia [10].

Bangladesh, a South Asian LwMIC with a large population of over 169 million, relies heavily on OOP financing for healthcare [11, 12]. Approximately 62% of the country's current health spending came from OOP sources in 2000, which increased to 74% in 2020 [13]. Several studies examined the level of CHE incidence in Bangladesh, focusing on its distribution across various equity strata, including household economic status and rural‒urban geographic differences [14–19]. A recent study [19] employed all the standard measurement methods defined in the Global UHC monitoring reports [20, 21], including the budget share method [9], the actual food expenditure method [9], the normative food expenditure method [8], the normative food, housing (rent), and utilities method [20, 22], to calculate the CHE incidence from nationally representative data. The study found that 3.9% to 12.5% of all Bangladeshi households in 2005 and 8.7% to 24.7% in 2016 incurred CHE. Notably, while the concentration of CHE across household economic status varied depending on the measurement approach, rural households consistently experienced higher CHE incidence than their urban counterparts, with the gap widening over time. The growing rural‒urban CHE disparity between 2005 and 2016 is unambiguous, as it was evident irrespective of the methods used [19]:

- Budget share method, 10% threshold: from 1.1%-point to 5.9%-point
- Actual food expenditure method, 40% threshold: from 1.3%-point to 5.0%-point
- Normative food expenditure method, 40% threshold: from 2.6%-point to 6.3%-point
- Normative food, rent, and utilities method: from 5.4%-point to 9.4%-point

Additionally, this rural‒urban disparity remained consistent across different samples, including all households (with or without illness), households with any disease as well as cardiovascular disease, and households with or without major noncommunicable diseases (NCDs) [14–18].

The extensive literature on financial protection in healthcare in low and middle-income countries (LMICs) has also persistently shown higher CHE incidence among rural households [23–31]. Despite such findings, only one study in China examined the main contributors to the rural‒urban CHE gap and found distributional differences in household income, household heads' educational attainment, and health awareness as the main contributors [31]. However, the study sample was restricted to households with NCDs only; therefore, generalisation of the study results was not possible for the population suffering from any illness.

Bangladesh's persistent and widening disparity in CHE incidence between rural and urban households underscores a crucial gap in ensuring overall financial protection in the country. This issue is particularly critical considering that over 103 million people in Bangladesh reside in rural areas, constituting 60% of the population [32]. The consistently higher incidence of CHE among this rural population than their urban counterparts hinders Bangladesh's progress toward its commitment to achieving UHC and the SDGs, which fundamentally emphasise the principle of 'leaving no one behind'. However, despite the acknowledged existence of these disparities, no research identifies the contributors to the rural‒urban CHE difference in Bangladesh. Addressing this research gap is crucial for developing informed policies to mitigate the CHE disparity and thereby move the nation closer to its objectives of safeguarding all individuals from the financial burdens of healthcare and advancing its broader commitments to UHC and the SDG agenda.

Therefore, this study aims to fill this research gap by identifying and quantifying the factors that significantly contribute to the difference in CHE incidences among rural and urban households, in order of importance, over time. To accomplish this, we conduct a multivariate decomposition analysis (for a nonlinear response model), which is extensively used to decompose group differences in health outcomes and healthcare utilisation [33–39]. Notably, our analysis considered rural and urban households seeking care for any disease or symptoms, including but not limited to NCDs.

The study findings on the key modifiable factors driving the rural‒urban disparities in CHE in Bangladesh aim to equip policymakers with crucial information to design targeted policy interventions to close this financial protection gap and advance the country's progress towards achieving UHC and, thus, the SDGs. Additionally, the insights from this study will have implications for other LMICs facing similar challenges, thus contributing to the broader literature on equity in financial protection.

## Methods

### Data source

This study utilises secondary data from the latest three rounds (2005, 2010, and 2016) of the nationally representative Bangladesh Household Income and Expenditure Survey (HIES). The Bangladesh Bureau of Statistics (BBS) conducts this cross-sectional survey with technical assistance from the World Bank approximately every five years, aiming to evaluate living standards and poverty levels by collecting information on household income, expenditure, consumption, savings, education, employment, health, and other relevant variables. In the 2005 and 2010 rounds of the HIES, a two-stage stratified random sampling method was employed, surveying 10,080 and 12,240 households, respectively [40, 41]. These surveys were designed to provide annual poverty estimates at the division level, the first-level administrative geographical partitions in Bangladesh, further subdivided into districts. The 2016 round adopted a stratified two-stage cluster sampling approach, surveying 46,076 households [42]. This survey was designed to provide reliable poverty estimates at the division and district levels. The present study analysed data from rural and urban households with no missing observations and at least one individual seeking care for an illness or symptom reported within the last 30 days preceding the surveys. The final sample sizes for the current analysis consist of 3,799 households (2,426 rural and 1,373 urban) for 2005, 6,233 households (4,266 rural and 1,967 urban) for 2010, and 22,016 households (15,645 rural and 6,371 urban) for 2016.

### Measurement of CHE

The method for measuring CHE influences equity in financial protection analyses [6, 43]. Given that a significant fraction of Bangladesh's population remained under the poverty line during the study period despite declining poverty rates over the years (from 40.0% in 2005 to 24.3% in 2016), it becomes crucial to employ a CHE measurement method that appropriately captures the healthcare expense burden on these economically vulnerable populations [42].

Studies comparing equity implications of the four CHE measurement methods mentioned in the Global UHC tracking reports showed that the traditional CHE measurement methods (budget share, actual food expenditure, and normative food expenditure) inadequately capture the financial burden of healthcare expenses on the poor population [43]. For instance, the non-normative methods (i.e., budget share and actual food expenditure), particularly the budget share method, tend to skew the financial burden towards wealthier households and under-represent it among low-income households. Conversely, the normative food, rent, and utilities method offers a more accurate measure of CHE across households of varying economic statuses [43]. This approach, by necessitating that poorer (wealthier) households consistently allocate a smaller (larger) fraction of their budget to be identified as incurring CHE, effectively rectifies the underestimation of the financial burdens of poor households inherent in the other three methods [5, 43]. Further details regarding this method's specific features and advantages over other methods, including equity implications, are discussed elsewhere [43, 44]. This method, increasingly adopted in recent LMIC financial protection literature, including Bangladesh, generates actionable evidence for policy-making [16, 19, 45, 46]. Consequently, we used the normative food, rent, and utilities method for our primary analysis, with results presented in the main text. Supplementary analyses using the three traditional methods are provided in the appendix (see Additional files 4, 5, and 6).

Following the normative food, rent, and utilities method, we considered a household to have incurred CHE if its OOP expenses were 40% or more of its CTP. Additionally, any OOP expense by "poor" households is considered catastrophic in this normative method. Because OOP expenses are measured relative to CTP in calculating CHE, the effective threshold (OOP as a percentage of total consumption expenditure) increases progressively with the household's economic status. The CTP of a household for healthcare is derived by subtracting its subsistence expenditure (SE) from its total consumption expenses. SE is estimated using expenditures on food, housing (rent), and utilities (electricity, gas/fuel, and water) by households in the 25th to 35th percentile of the distribution of consumption expenses per equivalent adult. In line with conventions in financial protection literature and to maintain international comparability, we utilised the World Health Organization’s (WHO) household equivalence scale in our computations [47]. We also conducted a parallel analysis using a Bangladesh-specific equivalence scale to ensure a context-specific assessment [48]. To calculate essential food spending, we excluded tobacco-related consumption and dining out [49]. Rents include actual rents for rented accommodations and imputed rent for owner-occupied housing. Households with total consumption expenditures below their standard SE level, resulting in a negative CTP or no CTP for healthcare, were classified as "poor." Therefore, mathematically, the CHE status of a household is defined as:

1 $$\begin{array}{cc}\begin{array}{cc}\mathrm{CHE}=1\;\mathrm{if}\;\left(\frac{\textrm{OOP}}{\text{CTP}}\geq0.4\right)\;\mathrm{or}&\left(\mathrm{CTP}<0\;\&\;\mathrm{OOP}>0\;\&\frac{\textrm{OOP}}{\text{CTP}}<0\right)\end{array}\\\mathrm{and}\\\mathrm{CHE}=0\;\mathrm{if}\;\left(0\leq\frac{\textrm{OOP}}{\text{CTP}}<0.4\right)\end{array}$$

The incidence of CHE is measured as the proportion of households incurring CHE among all households included in the analysis, i.e.,

2 $$\mathrm{CHE}\;\mathrm{incidence}=\frac{\mathrm{Number}\;\mathrm{of}\;\mathrm{households}\;\mathrm{incurring}\;\mathrm{CHE}}{\mathrm{Total}\;\mathrm{number}\;\mathrm{of}\;\mathrm{households}}$$

OOP expenses (as a separate variable and a component of total consumption expenditure) are derived from the health module of HIES and include direct healthcare expenditures (such as consultation, medicine, diagnosis, and hospital/clinic charges) but exclude indirect healthcare expenditures (such as transportation costs) [49, 50]. All expenses were adjusted for inflation using the consumer price index (CPI) and converted to US dollars based on the 2016 average exchange rate (BDT 78.468 = USD 1) [51, 52].

### Explanatory variables

An extensive literature review was conducted to identify variables included in studies examining the determinants of household CHE status. Furthermore, all three rounds of HIES datasets were examined to identify variables that could be considered for our study (Additional file 1). The selection of candidate variables was based on their availability in the HIES datasets. To address multicollinearity concerns, variables with a high variance inflation factor (VIF > 10) were identified (household head's age, marital status, religion, and employment status) and subsequently removed. The final set of explanatory variables included in the decomposition model encompassing household characteristics and household head characteristics are household economic status (consumption expenditure quintile), household head sex and education level, household size, number of earners in the household, presence of elderly members aged 60 years or above, presence of children aged five years or less, presence of chronically ill members, source of healthcare, and hospitalisation of household members. Additional file 2 provides the VIF values for the variables included in the final model.

### Statistical analysis

Descriptive analysis was carried out to evaluate distributional differences between rural and urban households. Bivariate analyses were conducted to examine whether the differences were statistically significant using design-adjusted chi-squared-alternative F tests for categorical variables (appropriate for complex survey data) and design-adjusted Wald tests for continuous variables. We then measured CHE incidences among rural and urban households for each survey year. As CHE is a nonlinear (binary) response variable, logit models were subsequently employed to estimate the effect of explanatory variables on CHE incidence. The specific regression model is as follows:

3 $${Y}_{j} =F\left({X}_{j}{\beta}_{j}\right)$$

where Y is the N × 1 vector of CHE incidence; X is an N x K matrix of the explanatory variables; β is the K × 1 vector of the regression coefficients. F denotes the logistic function, and subscript *j* represents urban or rural households.

Finally, the Oaxaca-Blinder multivariate decomposition technique adapted by Powers et al. for nonlinear response models was employed to quantify the contributions of explanatory variables to the rural‒urban difference in CHE incidence [53]. This technique divides the difference into two components: the explained part, E: reflecting differences due to varying characteristics or endowments between the two groups and the unexplained part, C: capturing differences due to variations in the effects of these characteristics, encompassing differences in returns, coefficients, or behavioural responses. In this context, the mean difference in CHE incidence between rural and urban households is defined as follows:

4 $$\overline{{Y}_{R}} - \overline{{Y}_{U}} =F\left({X}_{R} {\widehat{\beta }}_{R}\right) - F\left({X}_{U} {\widehat{\beta }}_{U}\right)$$

where subscript R is for rural households, and U is for urban households. The term $F\left({X}_{U} \widehat{{\beta }_{R}}\right)$ is then subtracted and added to arrive at the following:

5 $$\overline{{Y}_{R}} - \overline{{Y}_{U}} =\left[F\left({X}_{R} {\widehat{\beta }}_{R}\right) - F\left({X}_{U} {\widehat{\beta }}_{R}\right)\right] + \left[F\left({X}_{U} {\widehat{\beta }}_{R}\right) - F\left({X}_{U} {\widehat{\beta }}_{U}\right)\right]$$

6 $$=E +C$$

The two components, E and C, are derived from counterfactual comparisons between rural and urban households. E indicates the expected difference if rural households had the same distribution of covariates as urban households. Conversely, C reflects the expected difference if urban households had the same behavioural response to X as rural households. Notably, E and C are the weighted sums of ${E}_{k}$ and${C}_{k}$, respectively, where ${E}_{k}$ represents the contribution of the k^th^ covariate $(k=1, 2, 3, ....., K)$ to E; ${C}_{k}$ represents the contribution of the k^th^ covariate to C. A positive (negative) ${E}_{k}$ coefficient indicates the expected reduction (increase) in the rural‒urban CHE gap if rural households were equal to urban households in the distribution of${X}_{k}$. In contrast, a positive (negative) ${C}_{k}$ coefficient indicates an expected decrease (increase) in the rural‒urban CHE gap if rural families had the same behavioural responses or returns to risk as urban families.

We used the *mvdcmp* extension in Stata, selecting the logit model and incorporating survey weights for respective years [53]. We also utilized the normalization option, enabling the calculation of effects for all levels of categorical variables [54]. Statistical significance tests were conducted at 5% and 1% significance levels. We also decomposed rural‒urban disparities in CHE incidences using the budget share, actual food expenditure, and normative food expenditure methods (see Additional files 4, 5, and 6).

## Results

Table 1 presents the background characteristics and compositional differences between rural and urban households with members seeking care for any disease during the study period. Overall, most characteristics showed significant urban–rural differences. Specifically (in terms of the magnitude of rural‒urban differences), rural households consistently exhibited significantly (*p* value ≤ 0.01) higher prevalence of households with heads having no education (2005: 27.3%, 2010: 22.7%, 2016: 12.0%), households in the lowest consumption quintile (2005: 11.4%, 2010: 11.6%, 2016: 12.0%), reliance on informal healthcare sources such as pharmacies, self-treatment, homoeopathy, ayurveda, and other traditional and spiritual healers (2005: 14.2%, 2010: 8.1%, 2016: 8.8%), and households with elderly members (2005: 6.8%, 2010: 6.4%, 2016: 6.5%) than their urban counterparts. Conversely, households in rural areas invariably had a significantly (p value ≤ 0.01) lower prevalence of the highest quintile households (2005: -20.9%, 2010: -20.5%, 2016: -17.5%), households with heads having secondary or above-level education (2005: -21.6%, 2010: -16.5%, 2016: -15.4%), and those having a household size of three to four (2005: -7.7%, 2010: -6.8%, 2016: -4.7%) than urban households. The magnitude of the rural‒urban compositional difference across all the above characteristics generally declined between 2005 and 2016. Notably, while there was no significant rural‒urban difference (*p* value > 0.05) in the proportion of households with chronically ill individuals in 2005 (1.2%) and 2010 (1.4%), a substantial gap emerged in 2016 (9.0%, *p* value ≤ 0.01).

**Table 1:** Compositional difference of background characteristics between rural and urban households with members seeking care for any disease (%)

Characteristics	2005					2010					2016				
	Rural (*n* = 2,426)		Urban (*n* = 1,373)		Difference	Rural (*n* = 4,266)		Urban (*n* = 1,967)		Difference	Rural (*n* = 15,645)		Urban (*n* = 6,371)		Difference
Consumption expenditure quintile															
Lowest	18.2	(0.8)	6.8	(0.6)	11.4**	19.9	(0.9)	8.3	(0.8)	11.6**	19.6	(0.6)	7.6	(0.7)	12.0**
2nd	21.4	(0.8)	11.5	(0.9)	10.0**	21.7	(0.7)	13.4	(1.0)	8.3**	21.7	(0.5)	13.7	(1.1)	8.0**
3rd	21.7	(0.8)	15.8	(1.2)	5.9**	20.9	(0.6)	16.7	(1.2)	4.2**	21.1	(0.5)	18.4	(1.3)	2.7
4th	20.7	(0.8)	27.1	(1.6)	-6.4**	20.8	(0.7)	24.3	(1.6)	-3.6*	19.8	(0.6)	25.0	(1.4)	-5.2**
Highest	18.0	(0.8)	38.9	(1.7)	-20.9**	16.7	(0.8)	37.2	(2.3)	-20.5**	17.8	(0.6)	35.3	(1.8)	-17.5**
Female household head	10.2	(0.6)	8.3	(1.0)	1.9	13.0	(0.6)	10.7	(0.9)	2.3*	12.2	(0.4)	11.1	(0.7)	1.1
Education of household head															
No education	61.4	(1.0)	34.1	(1.6)	27.3**	58.0	(1.0)	35.3	(1.8)	22.7**	43.7	(0.8)	31.8	(1.2)	12.0**
Below secondary	28.7	(0.9)	34.5	(1.7)	-5.8**	31.0	(0.9)	37.2	(1.8)	-6.2**	46.6	(0.7)	43.2	(1.2)	3.4*
Secondary or above	9.8	(0.6)	31.4	(1.7)	-21.6**	11.0	(0.6)	27.5	(2.1)	-16.5**	9.6	(0.4)	25.0	(1.5)	-15.4**
Household size															
1–2 members	6.2	(0.5)	4.2	(0.7)	1.9*	8.3	(0.5)	7.9	(0.9)	0.4	10.7	(0.3)	10.6	(0.8)	0.1
3–4 members	35.2	(1.0)	43.0	(1.8)	-7.7**	40.5	(0.9)	47.3	(1.7)	-6.8**	49.4	(0.5)	54.1	(0.9)	-4.7**
5 or more members	58.6	(1.0)	52.8	(1.8)	5.8**	51.2	(0.9)	44.8	(1.9)	6.3**	39.9	(0.7)	35.4	(1.0)	4.5**
Earners^a^	1.4	(0.0)	1.5	(0.0)	-0.1**	1.3	(0.0)	1.4	(0.0)	-0.1**	1.2	(0.0)	1.3	(0.0)	-0.1**
Presence of elderly household member(s)	30.4	(0.9)	23.6	(1.4)	6.8**	30.8	(0.8)	24.5	(1.6)	6.4**	29.1	(0.5)	22.6	(1.2)	6.5**
Presence of children under five years	51.3	(1.0)	46.5	(1.8)	4.8*	43.5	(0.8)	40.8	(1.7)	2.7	40.8	(0.7)	42.2	(1.4)	-1.4
Presence of household member(s) with chronic illness	51.5	(1.0)	50.4	(1.8)	1.2	53.9	(1.1)	52.6	(2.2)	1.4	58.5	(0.8)	49.5	(1.8)	9.0**
Source of healthcare															
Public only	6.8	(0.5)	9.9	(1.0)	-3.1**	9.6	(0.6)	12.7	(1.6)	-3.1*	10.8	(0.5)	11.6	(0.7)	-0.9
Private only	32.7	(0.9)	42.7	(1.7)	-10.0**	34.9	(1.5)	40.1	(1.8)	-5.1*	19.9	(0.6)	28.8	(1.5)	-8.9**
Informal ^b^ only	48.9	(1.0)	34.7	(1.6)	14.2**	44.6	(1.6)	36.6	(1.9)	8.1**	54.0	(0.8)	45.2	(1.6)	8.8**
Public & private	0.8	(0.2)	2.1	(0.5)	-1.3**	2.4	(0.3)	2.8	(0.4)	-0.4	2.2	(0.2)	3.3	(0.5)	-1.1*
Public & informal	2.3	(0.3)	1.4	(0.3)	0.9*	2.1	(0.2)	1.8	(0.4)	0.3	5.4	(0.4)	4.0	(0.4)	1.4*
Private & informal	8.2	(0.6)	8.6	(1.0)	-0.4	6.1	(0.5)	5.8	(0.7)	0.2	6.9	(0.3)	6.4	(0.7)	0.5
Public, private & informal	0.3	(0.1)	0.5	(0.3)	-0.2	0.3	(0.1)	0.3	(0.1)	0.0	0.9	(0.1)	0.7	(0.1)	0.2
Hospitalization of household members	3.4	(0.4)	5.1	(0.8)	-1.7*	4.4	(0.4)	4.7	(0.6)	-0.3	10.8	(0.5)	12.3	(0.7)	-1.6

Adjusted with survey weights; Difference = Rural—Urban; numbers in parentheses are standard errors

Categorical variables are tested for differences using a design-based F-test (equivalent to a chi-squared test but appropriate for complex survey data). For the only continuous variable, number of earners, we test the equality of survey design-adjusted year-specific means (adjusted Wald test)

^a^ Expressed in average number per household

^b^ Includes healthcare sought from pharmacy salespersons, non-qualified doctors, homoeopaths, ayurveda/kabiraj/hekim, other traditional/spiritual healers, self-treatment, and others

^*^ p ≤ 0.05

^**^ p ≤ 0.01

In terms of OOP healthcare expenditures, as shown in Table 2, rural households spent USD 190.24 in 2005, significantly (*p* < 0.01) less than their urban counterparts, who spent USD 286.88. By 2016, rural households experienced approximately a 100% growth in OOP expenses to USD 370.18. Conversely, urban households witnessed a 44% increase in OOP expenses to USD 412.72 over the same period, albeit with fluctuation in 2010 (USD 271.35).

**Table 2:** Mean annual out-of-pocket (OOP) expenditures (USD) by area of residence

Year	Rural		Urban		Difference
	Mean	Std. Err	Mean	Std. Err	
2005	190.24	(10.3)	286.88	(22.9)	-96.63**
2010	280.78	(24.7)	271.35	(21.5)	9.43
2016	370.18	(10.8)	412.72	(21.3)	-42.54

OOP expenditures are adjusted for inflation using the consumer price index (CPI) and converted to US dollars based on the 2016 average exchange rate (USD 1 = BDT 78.468); Difference = Rural OOP expenditure—Urban OOP expenditure; Std. Err. = standard error; Equality of mean OOP expenses are tested using adjusted Wald test

^*^ *p* ≤ 0.05

^**^ *p* ≤ 0.01

Table 3 presents CHE incidences among rural and urban households over the years and the decomposition of the differences. Panel A shows that while the CHE incidence among urban households remained relatively stable between 2005 (15.11%) and 2016 (15.01%), with a dip in 2010 (11.80%), the incidence in rural households consistently increased from 24.85% in 2005 to 25.74% in 2010 and 27.91% in 2016. These rural rates significantly (p value ≤ 0.01) exceeded the urban rates by 9.74%-, 13.94%-, and 12.90%-points in 2005, 2010, and 2016, respectively.

**Table 3:** Aggregate and detailed decomposition of rural‒urban differences in catastrophic health expenditure (CHE) incidence

	Panel A: CHE incidence by household residence and aggregate decomposition of CHE difference											
	2005				2010				2016			
	Coefficient	Std. Err	Percent		Coefficient	Std. Err	Percent		Coefficient	Std. Err	Percent	
CHE incidence												
Rural	0.2485	(0.0086)			0.2574	(0.0098)			0.2791	(0.0066)		
Urban	0.1511	(0.0114)			0.1180	(0.0097)			0.1501	(0.0088)		
Total difference	0.0974**	0.0122	100.00		0.1394**	0.0091	100.00		0.1290**	0.0070	100.00	
Difference due to characteristics	0.0856**	0.0061	87.93		0.0842**	0.0037	60.44		0.0791**	0.0027	61.33	
Difference due to coefficients	0.0118	0.0131	12.07		0.0551**	0.0093	39.56		0.0499**	0.0071	38.67	
	Panel B: Detailed decomposition: Difference due to characteristics											
Characteristics	2005				2010				2016			
	Coefficient	Std. Err	Percent		Coefficient	Std. Err	Percent		Coefficient	Std. Err	Percent	
Consumption expenditure quintile				**84.91**				**50.09**				**55.20**
Lowest	0.0485**	(0.0041)	49.82		0.0504**	(0.0024)	36.16		0.0434**	(0.0011)	33.61	
2nd	0.0000	(0.0018)	0.04		-0.0028*	(0.0012)	-2.02		-0.0008	(0.0007)	-0.61	
3rd	-0.0072**	(0.0015)	-7.40		-0.0052**	(0.0007)	-3.76		-0.0021**	(0.0002)	-1.66	
4th	0.0098*	(0.0016)	10.10		0.0061**	(0.0006)	4.39		0.0071**	(0.0005)	5.47	
Highest	0.0315**	(0.0047)	32.35		0.0214**	(0.0033)	15.32		0.0237**	(0.0016)	18.39	
Female household head	0.0004	(0.0006)	0.39	**0.39**	0.0004	(0.0005)	0.28	**0.28**	-0.0003	(0.0002)	-0.20	**-0.20**
Education of household head				**35.30**				**15.89**				**12.23**
No education	0.0182**	(0.0044)	18.70		0.0127**	(0.0027)	9.09		0.0057**	(0.0008)	4.40	
Below secondary	-0.0007	(0.0011)	-0.68		-0.0002	(0.0008)	-0.12		0.0005*	(0.0002)	0.39	
Secondary or above	0.0168**	(0.0057)	17.28		0.0096**	(0.0030)	6.92		0.0096**	(0.0015)	7.44	
Household size				**-2.75**				**-0.88**				**-2.35**
1–2 members	0.0022**	(0.0005)	2.28		0.0004**	(0.0001)	0.28		0.0001**	(0.0000)	0.11	
3–4 members	0.0010	(0.0012)	1.06		0.0020**	(0.0008)	1.41		0.0007**	(0.0003)	0.54	
5 or more members	-0.0059**	(0.0012)	-6.09		-0.0036**	(0.0009)	-2.57		-0.0039**	(0.0004)	-3.00	
Number of earners	0.0011	(0.0014)	1.13	**1.13**	0.0037**	(0.0010)	2.62	**2.62**	0.0024**	(0.0009)	1.87	**1.87**
Presence of elderly household member(s)	-0.0010	(0.0016)	-1.06	**-1.06**	-0.0004	(0.0011)	-0.28	**-0.28**	0.0020**	(0.0006)	1.56	**1.56**
Presence of children under five years	0.0026**	(0.0010)	2.68	**2.68**	0.0011*	(0.0004)	0.77	**0.77**	-0.0003*	(0.0001)	-0.23	**-0.23**
Presence of household member(s) with chronic illness	0.0002	(0.0002)	0.17	**0.17**	0.0005	(0.0003)	0.33	**0.33**	0.0118**	(0.0008)	9.14	**9.14**
Source of healthcare				**-28.00**				**-7.69**				**-13.58**
Public only	0.0023	(0.0013)	2.31		0.0020*	(0.0009)	1.42		0.0006**	(0.0001)	0.48	
Private only	0.0077**	(0.0028)	7.88		0.0017	(0.0011)	1.18		-0.0012	(0.0010)	-0.96	
Informal only	-0.0355**	(0.0047)	-36.46		-0.0142**	(0.0018)	-10.17		-0.0162**	(0.0009)	-12.58	
Public & private	-0.0014	(0.0011)	-1.38		-0.0002	(0.0001)	-0.16		-0.0010**	(0.0002)	-0.77	
Public & informal	0.0003	(0.0004)	0.30		0.0001	(0.0002)	0.04		-0.0001	(0.0003)	-0.10	
Private & informal	-0.0002	(0.0002)	-0.17		0.0000	(0.0001)	0.03		0.0003**	(0.0001)	0.22	
Public, private & informal	-0.0005*	(0.0002)	-0.48		0.0000*	(0.0000)	-0.03		0.0002**	(0.0001)	0.13	
Hospitalization of household members	-0.0047**	(0.0007)	-4.84	**-4.84**	-0.0010**	(0.0001)	-0.70	**-0.70**	-0.0030**	(0.0002)	-2.33	**-0.70**
Characteristics	Panel C: Detailed decomposition: Difference due to coefficients											
	2005				2010				2016			
	Coefficient	Std. Err	Percent		Coefficient	Std. Err	Percent		Coefficient	Std. Err	Percent	
Consumption expenditure quintile				**8.45**				**3.77**				**3.40**
Lowest	-0.0027	(0.0017)	-2.72		-0.0016	(0.0012)	-1.13		-0.0026**	(0.0009)	-1.97	
2nd	0.0007	(0.0017)	0.69		-0.0002	(0.0020)	-0.14		-0.0018	(0.0016)	-1.43	
3rd	-0.0007	(0.0023)	-0.67		0.0035	(0.0031)	2.53		0.0087**	(0.0023)	6.74	
4th	0.0084	(0.0057)	8.64		-0.0070	(0.0040)	-5.02		-0.0004	(0.0036)	-0.29	
Highest	0.0024	(0.0058)	2.51		0.0105	(0.0058)	7.53		0.0005	(0.0048)	0.35	
Female household head	-0.0008	(0.0021)	-0.80	**-0.80**	-0.0016	(0.0024)	-1.13	**-1.13**	0.0017	(0.0021)	1.35	**1.35**
Education of household head				**-0.02**				**-1.52**				**0.14**
No education	0.0002	(0.0041)	0.18		-0.0039	(0.0044)	-2.81		-0.0004	(0.0034)	-0.28	
Below secondary	-0.0004	(0.0039)	-0.37		-0.0048	(0.0044)	-3.46		0.0006	(0.0043)	0.46	
Secondary or above	0.0002	(0.0046)	0.17		0.0066	(0.0044)	4.75		-0.0001	(0.0040)	-0.04	
Household size				**-13.07**				**2.33**				**2.14**
1–2 members	0.0011	(0.0013)	1.15		-0.0006	(0.0013)	-0.45		0.0005	(0.0015)	0.41	
3–4 members	-0.0004	(0.0063)	-0.36		0.0053	(0.0056)	3.80		0.0116*	(0.0049)	9.00	
5 or more members	-0.0135	(0.0117)	-13.86		-0.0014	(0.0057)	-1.02		-0.0094*	(0.0043)	-7.27	
Number of earners	-0.0032	(0.0134)	-3.30	**-3.30**	-0.0126	(0.0146)	-9.03	**-9.03**	0.0101	(0.0134)	7.86	**7.86**
Presence of elderly household member(s)	0.0022	(0.0042)	2.30	**2.30**	0.0048	(0.0041)	3.45	**3.45**	-0.0019	(0.0036)	-1.46	**-1.46**
Presence of children under five years	0.0151	(0.0117)	15.54	**15.54**	0.0079	(0.0072)	5.65	**5.65**	0.0036	(0.0058)	2.75	**2.75**
Presence of household member(s) with chronic illness	0.0117	(0.0092)	11.99	**11.99**	0.0107	(0.0084)	7.64	**7.64**	0.0032	(0.0078)	2.47	**2.47**
Source of healthcare				**-19.99**				**-17.17**				**0.93**
Public only	-0.0049	(0.0036)	-5.03		-0.0009	(0.0031)	-0.66		0.0005	(0.0020)	0.40	
Private only	-0.0171	(0.0114)	-17.57		-0.0192*	(0.0079)	-13.81		0.0036	(0.0041)	2.77	
Informal only	-0.0011	(0.0068)	-1.09		-0.0031	(0.0071)	-2.25		-0.0014	(0.0058)	-1.08	
Public & private	-0.0002	(0.0007)	-0.23		0.0001	(0.0010)	0.04		0.0002	(0.0010)	0.13	
Public & informal	0.0003	(0.0005)	0.34		0.0020*	(0.0010)	1.41		0.0013	(0.0010)	1.03	
Private & informal	0.0033	(0.0026)	3.35		-0.0026	(0.0014)	-1.89		-0.0030	(0.0017)	-2.29	
Public, private & informal	0.0002	(0.0003)	0.24		0.0000	(0.0002)	-0.01		0.0000	(0.0003)	-0.03	
Hospitalization of household members	-0.0003	(0.0013)	-0.26	**-0.26**	-0.0011	(0.0013)	-0.77	**-0.77**	-0.0019	(0.0021)	-1.46	**-1.46**
Constant	0.0110	(0.0257)	11.25	**11.25**	0.0646*	(0.0280)	46.37	**46.37**	0.0265	(0.0222)	20.57	**20.57**

CHE incidences are calculated using the normative food, rent, and utilities method at a 40% threshold (using the WHO household equivalence scale); Std. Err. = standard error

^*^ *p* ≤ 0.05

^**^ *p* ≤ 0.01

According to the aggregate decomposition of rural‒urban differences in CHE incidences (Panel A), most of the rural‒urban CHE differences can be attributed to differences in characteristics, endowment, or compositional factors (i.e., the explained part), which invariably accounted for a significant (*p* value ≤ 0.01) proportion: 87.93% in 2005, declining to 60.44% in 2010 and 61.33% in 2016. Consequently, the remaining portion of the predicted difference (the unexplained part), 12.07% in 2005 (*p* value > 0.05), increasing to 39.56% (*p* value ≤ 0.01) in 2010 and 38.67% (*p* value ≤ 0.01) in 2016, is either due to behavioural differences captured by covariate coefficients or is unexplained.

Panel B and C present a detailed decomposition of the aggregate rural‒urban difference in CHE, quantifying the contributions of each covariate and their levels to the differences attributed to characteristics and coefficients, respectively. In Panel B, examinations of the contribution of each covariate (consisting of the contributions of all levels) shows that household economic status consistently accounts for the largest contribution (2005: 84.91%, 2010: 50.09%, 2016: 55.20%) to the overall difference, followed by household head educational attainment (2005: 35.30%, 2010: 15.89%, 2016: 12.23%) and the source of healthcare (2005: -28.00%, 2010: -7.69%, 2016: -13.98%).

**Fig. 1:**
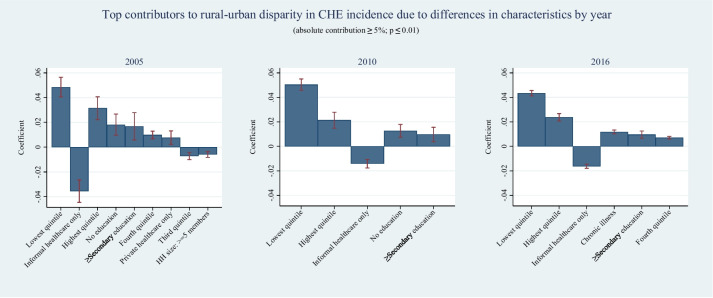
Covariate categories ranked by their absolute contribution (≥ 5%) to the disparity in rural‒urban CHE due to characteristic differences

At a more detailed level of analysis, when considering each category of the covariates separately, the primary categories consistently contributing significantly (p value ≤ 0.01) to the CHE gaps were the rural‒urban composition disparities in the lowest consumption quintile (2005: 49.82%, 2010: 36.16%, 2016: 33.61%), highest consumption quintile (2005: 32.35%, 2010: 15.32%, 2016: 18.39%), and exclusive reliance on informal healthcare sources (2005: -36.46%, 2010: -10.17%, 2016: -12.58%). These covariate categories remained the top three contributors, although their (absolute) magnitudes declined between 2005 and 2016. In 2005 and 2010, the subsequent significant (p value ≤ 0.01) contributors were the composition differences in households headed by individuals with no formal education (18.70% and 9.09%, respectively) and those with secondary or higher education (17.28% and 6.92%, respectively), followed by the contribution of the fourth consumption quintile (10.10% and 4.39%, respectively). Notably, the presence of chronically ill individuals in households emerged as a particularly influential factor in 2016, contributing (9.14%, *p* value ≤ 0.01) significantly to the difference and surpassing the geographic areawise difference in educational attainment of household heads. Additionally, in 2016, the relative importance of households with heads lacking education declined substantially (to 4.40%, p value ≤ 0.01), being outranked by the contribution from rural‒urban compositional differences in secondary or higher-level education of household heads (7.44%, *p* value ≤ 0.01) and the fourth quintile (5.47%, *p* value ≤ 0.01). Figure 1 illustrates the top contributing covariate categories by year.

The difference due to coefficient estimates in Panel C yielded large standard errors (compared to the standard errors in Panel B), so the contributions of only a few covariate levels were statistically significant. The intercepts primarily drove the increasing contribution of characteristic effects to CHE differences in recent periods (2010: 46.37%, *p* value ≤ 0.05; 2016: 20.57%, p value > 0.05), yet the contribution was significant in 2010 but not in 2016.

Utilising the Bangladesh household equivalence scale, the differences in CHE incidences between rural and urban households were marginally higher compared to those when using the WHO equivalence scale (2005: 11.21%- vs 9.74%-point; 2010: 14.40%- vs 13.94%-point; 2016: 13.16%- vs 12.90%-point). Regardless of the scale difference, the main contributors to the rural‒urban CHE disparity—household economic status (2005: 80.16%, 2010: 49.42%, 2016: 54.70%), education level of the household head (2005: 30.72%, 2010: 13.95%, 2016: 10.19%), and source of healthcare (2005: -23.50%, 2010: -7.34%, 2016: -12.90%)—remained consistent (see Additional file 3).

When applying the traditional methods, these factors maintained their consistency in their ranking and nature (positive or negative) of contribution for the normative food expenditure method, albeit with slightly lower contributions than the normative food, rent, and utilities method (see Additional file 4). In contrast, the budget share and actual food expenditure methods demonstrated a negative contribution (except for the latter method in 2016) of rural‒urban differences in economic status, particularly in the proportion of households in the lowest consumption quintile (see Additional files 5 and 6).

## Discussion

The rural‒urban disparity in CHE incidence is a well-documented challenge in LMICs, including Bangladesh, hindering financial protection and achieving the UHC target of the UN SDGs. This study is the first to identify key drivers of the rural‒urban differences in CHE incidence and their evolution in Bangladesh.

Analysis of three rounds of nationally representative data between 2005 and 2016 shows an increase in Bangladesh’s rural‒urban gap in CHE incidence. Most of the differences are attributable to compositional differences in characteristics between rural and urban households, with household economic status consistently contributing the most to the overall difference, followed by educational attainment of household heads and the source of healthcare. Specifically, despite a decline in contributions during the study period, composition disparities in the lowest and highest consumption quintiles and exclusive reliance on informal healthcare sources were consistently the top covariate categories contributing significantly to the CHE gaps. Noticeably, the presence of individuals with chronic illness in households emerged as a significant factor in 2016, superseding the contributions of the rural‒urban composition differences in households with heads having secondary and higher education and no education.

The rural and urban CHE incidences in this study are higher than those reported by previous studies in Bangladesh [14, 15, 19]. This study's focus on healthcare-seeking households, a subset of all surveyed households, explains the elevated incidence rates. In contrast to prior research encompassing all surveyed households, regardless of household members' illness or healthcare-seeking behaviour, our study employs a more focused denominator, including only households that actively engage with the healthcare system. This specific methodological approach naturally led to higher CHE incidence findings. Nevertheless, the result that the rural‒urban CHE gap in 2016 was wider than in 2005, corroborating existing evidence (but again with higher differences), poses a challenge in achieving financial protection against ill health and UHC and, thus, attaining the SDGs [19]. Further investigation revealed that CTP for healthcare increased for all households between 2005 and 2016, possibly brought about by the country’s sustained economic growth, among other factors. However, rural households experienced considerably faster growth in OOP expenses than urban households, both in absolute terms and relative to CTP. Consequently, more rural households incurred CHE, widening the rural‒urban CHE gap. The faster growth in OOP expenses among rural households may be due to increased healthcare utilisation driven by higher CTP and compounded by increased morbidity, including chronic illness, as found in the current study and previous research in Bangladesh [17, 19].

The primary finding of this study is that differences in characteristics significantly account for a substantial portion of the rural‒urban CHE disparities despite the increased contribution of the unexplained part (difference due to coefficients) over time. This finding highlights the potential to close a considerable portion of the CHE gap by shifting the distribution of the key contributing covariates in favour of rural households.

The study found household economic status to consistently have the largest positive contribution to the rural‒urban CHE disparity, consistent with findings from China [31]. Specifically, differences in the proportions of households in the lowest and highest consumption quintiles between rural and urban areas were invariably among the top three contributors to the CHE gaps, with positive but declining contributions between 2005 and 2016. The concentration of the lowest quintile households in rural areas and the highest consumption quintile in urban areas, combined with the lower likelihood of households with higher economic status incurring CHE in both settings (see Additional file 7), contributed to the significant disparity in CHE between rural and urban areas. Bangladesh's progress in reducing the poverty rate (headcount ratio: from 40% in 2005 to 24.3% in 2016) and income inequality (Gini index: from 33.2 in 2005 to 32.4 in 2016) during the study period appears to have lowered the contribution of economic status in explaining the CHE gap [55, 56]. Nevertheless, the decomposition results for the latest period (2016) indicate that a reduction of a third of the CHE gap is still associated with lowering the proportion of the lowest quintile households to the urban level and closing nearly an additional fifth of the gap remains linked to increasing the proportion of the highest quintile households in rural areas to the urban level.

The source of healthcare, particularly the difference in the extent to which rural and urban households rely exclusively on informal healthcare, is the following crucial characteristic that significantly explains the rural‒urban CHE disparity in each study year. Unlike economic status, this characteristic exhibits a negative contribution coefficient, implying that aligning rural households' reliance on informal healthcare with relatively lower urban levels would widen the CHE gap further.

In Bangladesh and other developing countries, informal healthcare providers (typically having little or no officially recognised training to provide healthcare), primarily pharmacy salespersons, play a crucial role in healthcare provision [57–59]. Convenience, affordability, and sociocultural acceptability are commonly cited reasons for choosing informal providers [59]. Shortages of qualified healthcare professionals and the widespread availability of informal providers in rural areas drive rural residents of Bangladesh and other LMICs towards informal providers [58, 60, 61]. Previous studies have highlighted that formal healthcare institutions in rural Bangladesh, particularly public facilities such as Upazila Health Centers, are less service-ready than their urban counterparts [62]. Although Bangladesh has established an extensive network of public primary healthcare facilities in rural areas, healthcare workers are concentrated in urban tertiary facilities due to low retention of qualified professionals in rural settings [60, 63]. Official government data indicate high vacancy rates of medical doctors in rural public primary healthcare levels, ranging from 40–80% [63]. Additionally, the unavailability of essential medicines is a persistent issue in public facilities, especially in rural areas [62, 64]. Consequently, many rural residents rely on informal providers, mainly pharmacies, as their primary or sometimes the only healthcare source [57, 65, 66].

We found sole reliance on informal sources as a protective factor against CHE in rural and urban settings. Previous studies have also demonstrated that seeking care from informal caregivers is considerably more affordable, ranging from five to fifteen times less expensive than seeking care from formal providers in Bangladesh [59, 67]. The affordability of informal healthcare may also stem from informal providers typically not charging consultation fees and ordering clinical diagnostic tests. However, it can also result from people seeking care from these providers for generally nonsevere illnesses requiring inexpensive treatments [67, 68]. Thus, higher exclusive reliance of rural households on apparently cheaper informal care is a mitigating factor in the rural‒urban CHE gap. However, from 2005 to 2016, the compositional differences between rural and urban households shrank with higher growth in urban households' reliance on informal sources (primarily pharmacies, as further investigation revealed). Accordingly, the contribution of this characteristic difference to the rural‒urban CHE disparity in 2016 was lower in absolute terms compared to 2005.

While higher reliance of rural households on informal healthcare sources acts as a factor in mitigating the CHE gap, it is essential to recognise that informal providers often lack the proper training and equipment to deliver evidence-based, high-quality care [57]. Therefore, informal care, despite its accessibility and affordability, may not be as effective in managing or curing illnesses as medically trained formal providers [66]. Consequently, if health problems persist or aggravate, households may seek care from formal providers, resulting in increased healthcare spending and a higher risk of CHE in subsequent periods [59, 67]. Although this possibility is not examinable due to the cross-sectional nature of the data, we did observe that households seeking care from a mix of informal and formal sources were generally more likely to incur CHE than those seeking care from public (formal) sources only and, by implication, than those seeking care from informal sources only.

The emergence of individuals with chronic illness as a significant contributor to the CHE gap in the latest period is attributable to the proportion of rural households being markedly higher than urban households with this characteristic in that year. Previous evidence indicates an overall rise in NCD-affected households (including multiple NCDs) in Bangladesh between 2010 and 2016, with the proportion increasing among low-income households and declining among the highest-income group [16, 19]. Other research has shown that NCDs, such as prehypertension, hypertension, prediabetes, and diabetes, are not only prevalent among the urban wealthy but also among the poorest population in rural Bangladesh [69]. Since NCDs are chronic conditions requiring prolonged and costly treatment, households affected by NCDs are more likely to incur CHE, particularly low-income households with limited CTP for healthcare. Consequently, the significant rural‒urban difference in chronic illness prevalence becomes a critical determinant of the CHE gap, outranking education levels of household heads in the latest study period.

Bangladesh's overall improvement in adult literacy rate during the study period (from 53.5% in 2005 to 72.3% in 2016), accompanied by a reduction in illiterate persons and a declining urban–rural literacy gap, may have diminished the influence of household heads' education on the CHE disparity, especially when household heads had no formal education [70, 71]. However, the composition disparity in secondary or higher education of household head was still considerable in 2016 (surpassing that of no education). Consequently, its contribution remained substantial, associated with over 7% reduction in the rural‒urban CHE gap by increasing the proportion of rural households with secondary or higher educated heads at the urban level.

While the intercepts primarily drove the increasing contribution of the CHE difference due to characteristic effects in recent periods, their contribution was not consistently significant, particularly in 2016, suggesting that the effect differences (not composition differences) of unaccounted-for variables in the decomposition model were not significantly different between rural and urban households.

The counter-intuitive finding that rural‒urban economic status differences contribute negatively to CHE disparity when using budget share and actual food expenditure methods warrants explanation. This result suggests that equalising rural economic status with urban levels would widen the CHE gap. Such an outcome is most likely due to the limitations of these methods, particularly their tendency to underestimate CHE among low-income households and overestimate it among wealthier households [43]. Further investigation revealed that households with higher economic status are more likely to incur CHE when measured through these methods, than their lower-income counterparts. As a result, these methods suggest that an increase in income among rural low-income groups could lead to a rise in their CHE incidence, (erroneously) indicating a growing disparity. Therefore, carefully considering this evidence in policy-making is crucial to avoid misguided decisions.

Based on our findings, closing the rural‒urban CHE disparity calls for a multifaceted approach; the pivotal of these is improving the economic status of rural households. Measures aimed at increasing economic opportunities and alleviating poverty further in rural regions will contribute to narrowing income inequality between rural and urban areas. Such efforts would raise the CTP for healthcare among the rural poor population, subsequently reducing the incidence of CHE in these communities. This improvement would enhance financial protection for the entire population, bringing the country closer to achieving UHC and the SDGs. In this context, strengthening safety nets for low-income rural households is crucial in protecting them against CHE.

Various social safety net programs are in place in Bangladesh, primarily benefiting rural poor households [72]. Among these programs, cash allowances, food support, maternity allowances, and employment generation programs for the poorest outperform others in reaching the poor and vulnerable [73]. While studies affirm that these programs aid in poverty reduction and bolstering food security [74–76], their impact is presently restrained by challenges such as inconsistent coverage in poorer areas, inclusion and exclusion errors in beneficiary selection, and insufficient benefit amounts [72, 73, 77]. Expanding the coverage and benefits of these programs while ensuring accurate beneficiary targeting would be a potent way to alleviate rural poverty and realise its broader effect on reducing rural‒urban CHE disparity and advancing the country towards achieving UHC and fostering inclusive development goals.

Furthermore, the ‘*Shasthyo Shuraksha Karmasuchi*’, a fully publicly funded pilot social health protection scheme for below-poverty-line (BPL) households in three subdistricts, is a notable UHC strategy by the Bangladesh government. This scheme has demonstrated success in reducing OOP expenses and CHE incidence among its beneficiaries [78]. The scheme currently covers the costs of inpatient care and outpatient consultations for 78 categories of diseases, including NCDs [79]. Scaling up this scheme to include the country's entire BPL population and extending the benefits package to cover NCD medications would benefit the poor population in rural areas, who now increasingly suffer from chronic conditions [16]. Recognising the growing burden of NCDs, the government has established NCD corners in some rural primary care facilities at the subdistrict level (i.e., Upazila Health Complexes). However, these corners are still in the early stages of development [79]. It is crucial to ensure their complete functionality to effectively address the burden of chronic NCDs, the high cost of treatment associated with them, and the risk of CHE in rural areas.

Addressing the geographic imbalance in overall service readiness is essential. Critical is the availability of physicians and essential medicines in rural public facilities to encourage rural residents to seek evidence-based, rational care within the formal healthcare system. However, given the size and importance of the informal sector, particularly in rural areas, strategies should be developed to accommodate informal providers in the mainstream health system [57, 58]. To that end, the government should invest in informal providers' capacity development through training, making them competent in preventive care (such as positive lifestyle change advising, given their sociocultural acceptability) and some curative care (limited to treating common, uncomplicated health problems).

Also crucial is improving the education level of household heads in rural areas, which warrants emphasis on adult literacy programs in rural areas, particularly those with low literacy rates, such as the northern rural areas where the literacy rate significantly lags urban areas [80]. Such initiatives can catalyse a generational shift in educational attainment, as educated adults are more likely to prioritise their children's education, thereby elevating rural education levels (for children and eventually for the entire rural population, including adults). According to Grossman's human capital theory, individuals with higher education levels are more efficient producers of health [81]. Improving education in rural areas, including health awareness, is expected to enhance the rural population's health status, reduce disease prevalence, including chronic illnesses, and diminish OOP expenses and the rural‒urban CHE disparity.

Our study is primarily limited by its cross-sectional data structure, which only allows for the analysis of correlational associations. Therefore, it is essential to note that the study establishes links between differences in the rural‒urban distribution of covariates and variations in CHE incidence rather than identifying the specific causes of CHE among rural or urban households. Additionally, the sampling design for the Bangladesh HIES changed in 2016 after remaining consistent between 2005 and 2010. This change necessitated a cautious approach in our analysis. Instead of pooling data across years, we conducted separate analyses for each year, applying survey round-specific sampling weights. This strategy ensured the robustness of our findings and the validity of our comparative analysis while avoiding potential biases from pooling data from different surveys with varying sampling designs. Also, HIES includes self-reports of disease occurrence data, so the possibility of reporting errors cannot be ruled out. We acknowledge that CHE captures one of several aspects of financial protection. We chose CHE as our focus due to its wide recognition in the literature and by the SDGs framework as a key indicator of financial protection [5]. Other indicators, such as impoverishment due to OOP expenses, adoption of coping strategies, and forgoing care for financial reasons, also represent a lack of financial protection among a population.

Notwithstanding these limitations, to our knowledge, this study provides the first evidence of the factors significantly contributing to rural‒urban CHE disparity in Bangladesh and the LwMIC context. These findings are crucial for Bangladesh and similar LMICs, including those aiming to achieve inclusive and sustainable development goals like the African Agenda 2063 [82], as they strive towards the SDGs amidst challenges of higher healthcare-related financial burdens in rural areas. This knowledge can inform policy-making to address these issues and accelerate progress towards achieving UHC and broader developmental goals.

Given the persistent disparity in CHE incidence between rural and urban households in Bangladesh and the increasing (declining) contribution of differences due to coefficients (characteristics) to this disparity, it is crucial to continue monitoring this disparity and its determinants over time. Such assessments will aid in identifying any shifts in critical determinants and relevant policies, including whether behaviour change interventions are required to bridge the gap. In addition, expanding the scope of analysis to encompass the other dimensions of financial protection, as mentioned above, will offer a more comprehensive understanding of the contributors to financial protection disparity, guiding policy-making towards improving equity in overall financial protection and inclusive development. This recommendation is not only applicable to Bangladesh but also extends to other LMICs experiencing similar problems.

## Conclusion

In conclusion, this study finds persistently higher CHE incidence among rural households in Bangladesh than their urban counterparts between 2005 and 2016, which poses a challenge to achieving UHC and, thus, the UN SDGs. Despite declining over time, compositional differences in household characteristics primarily drive the CHE gap. Reducing the proportion of rural households in the lowest consumption quintile, those with chronic illness, and those with heads having no formal education while increasing the proportion in the highest consumption quintile, with secondary or higher educated heads, at the urban level are found to be significantly associated with closing the CHE disparity. However, reducing the proportion of rural households relying solely on informal healthcare providers at the urban level is associated with widening the CHE gap. Improving the economic status of rural households, implementing safety nets against healthcare consumption, and strengthening the public healthcare system in rural areas are crucial steps toward reducing rural‒urban disparities in CHE. Additionally, investing in education in rural areas and incorporating informal healthcare providers into the mainstream health system through capacity building could be essential strategies.

### Supplementary Information

**Additional file 1.** Explanatory variables identified from the literature review and available in Bangladesh Household Income and Expenditure Surveys 2005, 2010, and 2016.

**Additional file 2.** VIFs of the variables included in the decomposition model.

**Additional file 3.** Mean CHE incidence among rural and urban households; aggregate and detailed decomposition of the difference in CHE incidence between rural and urban areas: normative food, rent, and utilities method, 40% threshold (using Bangladesh equivalence scale).

**Additional file 4.** Mean CHE incidence among rural and urban households; aggregate and detailed decomposition of the difference in CHE incidence between rural and urban areas: normative food expenditure method, 40% threshold.

**Additional file 5.** Mean CHE incidence among rural and urban households; aggregate and detailed decomposition of the difference in CHE incidence between rural and urban households: budget share method, 10% threshold.

**Additional file 6.** Mean CHE incidence among rural and urban households; aggregate and detailed decomposition of the difference in CHE incidence between rural and urban households: actual food expenditure method, 40% threshold.

**Additional file 7.** Logit model of CHE incidence (normative food, rent, and utilities method, 40% threshold) among urban and rural households, adjusted for all covariates.

## Data Availability

The datasets generated/or analysed for the current study are available from the Bangladesh Bureau of Statistics, Statistics and Informatics Division, Government of Bangladesh upon subscription. (http://data.bbs.gov.bd/index.php/catalog/HIES/about).

## Abbreviations

BBS: Bangladesh Bureau of Statistics
BDT: Bangladeshi taka
BHFS: Bangladesh health facility survey
BPL: Below-poverty-line
CHE: Catastrophic health expenditure
CPI: Consumer price index
CTP: Capacity-to-pay
HIES: Household Income and Expenditure Survey
LMIC: Low- and middle-income country
LwMIC: Lower middle-income country
NCD: Noncommunicable disease
OOP: Out-of-pocket
SDG: Sustainable Development Goals
SE: Subsistence expenditure
SSK: Shasthyo Suroksha Karmasuchi
UHC: Universal Health Coverage
UN: United Nations
USD: United States dollar
VIF: Variance inflation factor
WHO: World Health Organization
