# Supplementary material for: Rural‒urban disparities in household catastrophic health expenditure in Bangladesh: a multivariate decomposition analysis
Source: Int J Equity Health. 2024 Feb 27;23:43. doi: 10.1186/s12939-024-02125-3 (PMC10898052; doi:10.1186/s12939-024-02125-3)
Supplement: Supplementary file 1 — Additional file 1. Explanatory variables identified from the literature review and available in Bangladesh Household Income and Expenditure Surveys 2005, 2010, and 2016. [file 12939_2024_2125_MOESM1_ESM.docx]

**Additional Table 1:** Explanatory variables identified from the literature review and available in Bangladesh Household Income and Expenditure Surveys 2005, 2010, and 2016

| **Variable** | **Reference** |
| --- | --- |
| **Household head’s characteristics** | |
| Gender | (Ahmed et al. 2022; Barasa et al. 2017; Bashir & Kishwar 2021; Dwivedi et al. 2021; Edeh 2022; Edoka et al. 2017; Fu et al. 2021; Ghimire et al. 2018; Han et al. 2018; Khan et al. 2017; Liu et al. 2021; Mohanty & Dwivedi 2021; Mulaga et al. 2021; Saksena et al. 2010; Sharma et al. 2023; Shokri et al. 2023; Thu Thuong et al. 2021; Zhao et al. 2020) |
|  |  |
| Age | (Barasa et al. 2017; Bashir & Kishwar 2021; Edoka et al. 2017; Fu 2022; Han et al. 2018; Khan et al. 2017; Liu et al. 2021; Mohanty & Dwivedi 2021; Mulaga et al. 2021; Sharma et al. 2023; Shokri et al. 2023; Thu Thuong et al. 2021; Zhao et al. 2020) |
|  |  |
| Marital status | (Bashir & Kishwar 2021; Dwivedi et al. 2021; Fu 2022; Fu et al. 2021; Liu et al. 2021; Sriram & Albadrani 2022; Thu Thuong et al. 2021; Zhao et al. 2020) |
|  |  |
| Education level | (Ahmed et al. 2022; Bashir & Kishwar 2021; Dwivedi et al. 2021; Edeh 2022; Edoka et al. 2017; Fu 2022; Fu et al. 2021; Ghimire et al. 2018; Han et al. 2018; Khan et al. 2017; Liu et al. 2021; Mohanty & Dwivedi 2021; Saksena et al. 2010; Sharma et al. 2023; Shokri et al. 2023; Zhao et al. 2020) |
|  |  |
| Employment/ earning status | (Barasa et al. 2017; Bashir & Kishwar 2021; Edeh 2022; Fu 2022; Ghimire et al. 2018; Mohanty & Dwivedi 2021; Sharma et al. 2023; Shokri et al. 2023; Thu Thuong et al. 2021; Zhao et al. 2020) |
|  |  |
| Religion | (Dwivedi et al. 2021; Edoka et al. 2017; Mohanty & Dwivedi 2021) |
|  |  |
| **Household characteristics** | |
| Economic status | (Ahmed et al. 2022; Barasa et al. 2017; Bashir & Kishwar 2021; Chen et al. 2023; Dwivedi et al. 2021; Edeh 2022; Edoka et al. 2017; Fu 2022; Fu et al. 2021; Ghimire et al. 2018; Han et al. 2018; Khan et al. 2017; Liu et al. 2021; Mohanty & Dwivedi 2021; Mulaga et al. 2021; Saksena et al. 2010; Sharma et al. 2023; Shokri et al. 2023; Sriram & Albadrani 2022; Thu Thuong et al. 2021; Zhao et al. 2020) |
|  |  |
| Geographic area of residence | (Ahmed et al. 2022; Dwivedi et al. 2021; Edeh 2022; Edoka et al. 2017; Han et al. 2018; Khan et al. 2017; Mohanty & Dwivedi 2021; Mulaga et al. 2021; Saksena et al. 2010; Sharma et al. 2023; Shokri et al. 2023; Sriram & Albadrani 2022; Zhao et al. 2020) |
|  |  |
| Household size | (Ahmed et al. 2022; Barasa et al. 2017; Bashir & Kishwar 2021; Chen et al. 2023; Dwivedi et al. 2021; Edeh 2022; Edoka et al. 2017; Fu 2022; Fu et al. 2021; Ghimire et al. 2018; Han et al. 2018; Khan et al. 2017; Liu et al. 2021; Mohanty & Dwivedi 2021; Mulaga et al. 2021; Sharma et al. 2023; Shokri et al. 2023; Sriram & Albadrani 2022; Thu Thuong et al. 2021; Zhao et al. 2020) |
|  |  |
| Number of earners | (Bashir & Kishwar 2021) |
|  |  |
| Presence of elderly members | (Ahmed et al. 2022; Barasa et al. 2017; Bashir & Kishwar 2021; Chen et al. 2023; Edeh 2022; Edoka et al. 2017; Fu 2022; Fu et al. 2021; Ghimire et al. 2018; Han et al. 2018; Liu et al. 2021; Mulaga et al. 2021; Saksena et al. 2010; Sharma et al. 2023; Shokri et al. 2023; Sriram & Albadrani 2022; Thu Thuong et al. 2021; Zhao et al. 2020) |
|  |  |
| Presence of children | (Ahmed et al. 2022; Bashir & Kishwar 2021; Chen et al. 2023; Edoka et al. 2017; Fu et al. 2021; Ghimire et al. 2018; Mulaga et al. 2021; Saksena et al. 2010; Sharma et al. 2023; Shokri et al. 2023; Sriram & Albadrani 2022; Thu Thuong et al. 2021; Zhao et al. 2020) |
|  |  |
| Presence of chronically ill members | (Ahmed et al. 2022; Barasa et al. 2017; Chen et al. 2023; Fu 2022; Ghimire et al. 2018; Han et al. 2018; Liu et al. 2021; Mulaga et al. 2021; Zhao et al. 2020) |
|  |  |
| **Healthcare utilization** | |
| Source of healthcare | (Ahmed et al. 2022; Edoka et al. 2017; Mulaga et al. 2021; Sriram & Albadrani 2022; Thu Thuong et al. 2021) |
|  |  |
| Hospitalization of household members | (Ahmed et al. 2022; Fu 2022; Fu et al. 2021; Liu et al. 2021; Mohanty & Dwivedi 2021; Mulaga et al. 2021; Shokri et al. 2023; Sriram & Albadrani 2022) |
|  |  |

**References:**

Ahmed S, Ahmed MW, Hasan MZ, et al. 2022. Assessing the incidence of catastrophic health expenditure and impoverishment from out-of-pocket payments and their determinants in Bangladesh: Evidence from the nationwide Household Income and Expenditure Survey 2016. *International Health*, **14**: 84-96.

Barasa EW, Maina T, Ravishankar N. 2017. Assessing the impoverishing effects, and factors associated with the incidence of catastrophic health care payments in Kenya. *International Journal for Equity in Health*, **16**: 1-14.

Bashir S, Kishwar S. 2021. Incidence and determinants of catastrophic health expenditures and impoverishment in Pakistan. *Public Health*, **197**: 42-47.

Chen M, Xu L, Si L, Wang Z, Jan S. 2023. Examining the level and distribution of catastrophic health expenditure from 2013 to 2018: A province-level study in China. *Economic Modelling*, **121**: 106233.

Dwivedi R, Pradhan J, Athe R. 2021. Measuring catastrophe in paying for healthcare: A comparative methodological approach by using National Sample Survey, India. *International Journal of Health Planning and Management*, **36**: 1887-1915.

Edeh HC. 2022. Exploring dynamics in catastrophic health care expenditure in Nigeria. *Health Economics Review*, **12**.

Edoka I, McPake B, Ensor T, Amara R, Edem-Hotah J. 2017. Changes in catastrophic health expenditure in post-conflict Sierra Leone: An Oaxaca-blinder decomposition analysis. *International Journal for Equity in Health*, **16**.

Fu X-z. 2022. The comparison of catastrophic health expenditure and its inequality between urban and rural households in China. *Health Economics Review*, **12**: 19.

Fu X-z, Sun Q-w, Sun C-q, Xu F, He J-j. 2021. Urban-rural differences in catastrophic health expenditure among households with chronic non-communicable disease patients: evidence from China family panel studies. *BMC Public Health*, **21**: 874.

Ghimire M, Ayer R, Kondo M. 2018. Cumulative incidence, distribution, and determinants of catastrophic health expenditure in Nepal: Results from the living standards survey. *International Journal for Equity in Health*, **17**.

Han SM, Rahman MM, Rahman MS, et al. 2018. Progress towards universal health coverage in Myanmar: a national and subnational assessment. *The Lancet Global Health*, **6**: e989-e997.

Khan JAM, Ahmed S, Evans TG. 2017. Catastrophic healthcare expenditure and poverty related to out-of-pocket payments for healthcare in Bangladesh—an estimation of financial risk protection of universal health coverage. *Health policy and planning*, **32**: 1102-1110.

Liu C, Liu Z-m, Nicholas S, Wang J. 2021. Trends and determinants of catastrophic health expenditure in China 2010–2018: a national panel data analysis. *BMC Health Services Research*, **21**: 526.

Mohanty SK, Dwivedi LK. 2021. Addressing data and methodological limitations in estimating catastrophic health spending and impoverishment in India, 2004–18. *International Journal for Equity in Health*, **20**: 1-18.

Mulaga AN, Kamndaya MS, Masangwi SJ. 2021. Examining the incidence of catastrophic health expenditures and its determinants using multilevel logistic regression in Malawi. *PLoS ONE*, **16**.

Saksena P, Xu K, Durairaj V. 2010. The drivers of catastrophic expenditure: outpatient services, hospitalization or medicines. *World health report*, **1**: 21.

Sharma J, Pavlova M, Groot W. 2023. Catastrophic health care expenditure and impoverishment in Bhutan. *Health Policy and Planning*, **38**: 228-238.

Shokri A, Bolbanabad AM, Rezaei S, Moradi G, Piroozi B. 2023. Has Iran achieved the goal of reducing the prevalence of households faced with catastrophic health expenditure to 1%?: A national survey. *Health Science Reports*, **6**: e1199.

Sriram S, Albadrani M. 2022. A study of catastrophic health expenditures in India-evidence from Nationally Representative Survey Data: 2014-2018. *F1000Research*, **11**.

Thu Thuong NT, Van Den Berg Y, Huy TQ, Tai DA, Anh BNH. 2021. Determinants of catastrophic health expenditure in Vietnam. *International Journal of Health Planning and Management*, **36**: 316-333.

Zhao Y, Oldenburg B, Mahal A, et al. 2020. Trends and socio-economic disparities in catastrophic health expenditure and health impoverishment in China: 2010 to 2016. *Tropical Medicine and International Health*, **25**: 236-247.
