# Supplementary material for: Rural‒urban disparities in household catastrophic health expenditure in Bangladesh: a multivariate decomposition analysis
Source: Int J Equity Health. 2024 Feb 27;23:43. doi: 10.1186/s12939-024-02125-3 (PMC10898052; doi:10.1186/s12939-024-02125-3)
Supplement: Supplementary file 2 — Additional file 2. VIFs of the variables included in the decomposition model. [file 12939_2024_2125_MOESM2_ESM.docx]

**Additional Table 2:** Variance inflation factors (VIFs) of the variables included in the decomposition model

| **Variable** | **VIF** | | |
| --- | --- | --- | --- |
|  | **2005** | **2010** | **2016** |
| Consumption expenditure quintile |  |  |  |
| 2nd | 2.12 | 2.02 | 2.00 |
| 3rd | 2.31 | 2.11 | 2.10 |
| 4th | 2.63 | 2.30 | 2.27 |
| Highest | 3.15 | 2.63 | 2.68 |
|  |  |  |  |
| Female household head | 1.19 | 1.20 | 1.20 |
|  |  |  |  |
| Education of household head |  |  |  |
| Below secondary | 1.72 | 1.70 | 2.20 |
| Secondary or above | 1.60 | 1.51 | 1.52 |
|  |  |  |  |
| Household size |  |  |  |
| 3-4 members | 5.29 | 4.84 | 5.01 |
| 5 or more members | 9.42 | 7.11 | 4.97 |
|  |  |  |  |
| Number of earners | 4.29 | 4.27 | 4.21 |
|  |  |  |  |
| Presence of elderly household member(s) | 1.53 | 1.51 | 1.50 |
|  |  |  |  |
| Presence of children under five years | 2.29 | 2.01 | 1.97 |
|  |  |  |  |
| Presence of household member(s) with chronic illness | 2.17 | 2.25 | 2.44 |
|  |  |  |  |
| Source of healthcare |  |  |  |
| Private only | 4.24 | 3.48 | 2.54 |
| Informal only | 4.93 | 3.87 | 4.18 |
| Public & private | 1.11 | 1.20 | 1.19 |
| Public & informal | 1.21 | 1.14 | 1.34 |
| Private & informal | 1.80 | 1.46 | 1.49 |
| Public, private & informal | 1.05 | 1.03 | 1.07 |
|  |  |  |  |
| Hospitalization of household members | 1.08 | 1.10 | 1.19 |
|  |  |  |  |
| **Mean VIF** | **2.76** | **2.44** | **2.35** |
