# Supplementary material for: Rural‒urban disparities in household catastrophic health expenditure in Bangladesh: a multivariate decomposition analysis
Source: Int J Equity Health. 2024 Feb 27;23:43. doi: 10.1186/s12939-024-02125-3 (PMC10898052; doi:10.1186/s12939-024-02125-3)
Supplement: Supplementary file 3 — Additional file 3. Mean CHE incidence among rural and urban households; aggregate and detailed decomposition of the difference in CHE incidence between rural and urban areas: normative food, rent, and utilities method, 40% threshold (using Bangladesh equivalence scale). [file 12939_2024_2125_MOESM3_ESM.docx]

**Additional Table 3**: Rural-urban differences in catastrophic health expenditure (CHE) incidence, using the Bangladesh equivalence scale

|  | Panel A: CHE incidence by household residence and aggregate decomposition of CHE difference | | | | | | | | | | | |
| --- | --- | --- | --- | --- | --- | --- | --- | --- | --- | --- | --- | --- |
|  | 2005 | | | | 2010 | | | | 2016 | | | |
|  | Coefficient | Std. Err. | Percent | | Coefficient | Std. Err. | Percent | | Coefficient | Std. Err. | Percent | |
| CHE incidence |  |  |  |  |  |  |  |  |  |  |  |  |
| Rural | 0.2571 | (0.0088) |  |  | 0.2645 | (0.0101) |  |  | 0.2849 | (0.0066) |  |  |
| Urban | 0.1450 | (0.0110) |  |  | 0.1205 | (0.0098) |  |  | 0.1532 | (0.0096) |  |  |
|  |  |  |  |  |  |  |  |  |  |  |  |  |
| Total difference | 0.1121** | (0.0121) | 100.00 | | 0.1440** | (0.0094) | 100.00 | | 0.1316** | (0.0072) | 100.00 | |
|  |  |  |  | |  |  |  | |  |  |  | |
| Difference due to characteristics | 0.0905** | (0.0061) | 80.75 | | 0.0847** | (0.0038) | 58.80 | | 0.0783** | (0.0027) | 59.48 | |
| Difference due to coefficients | 0.0216 | (0.0129) | 19.25 | | 0.0593** | (0.0096) | 41.20 | | 0.0533** | (0.0074) | 40.52 | |
|  |  |  |  |  |  |  |  |  |  |  |  |  |
|  | Panel B: Detailed decomposition: Difference due to characteristics | | | | | | | | | | | |
| Characteristics | 2005 | | | | 2010 | | | | 2016 | | | |
|  | Coefficient | Std. Err. | Percent | | Coefficient | Std. Err. | Percent | | Coefficient | Std. Err. | Percent | |
| Consumption expenditure quintile |  |  |  | **80.16** |  |  |  | **49.42** |  |  |  | **54.70** |
| Lowest | 0.0578** | (0.0045) | 51.59 |  | 0.0498** | (0.0023) | 34.59 |  | 0.0429** | (0.0011) | 32.61 |  |
| 2nd | -0.0010 | (0.0020) | -0.89 |  | -0.0023 | (0.0013) | -1.57 |  | 0.0004 | (0.0007) | 0.33 |  |
| 3rd | -0.0078** | (0.0013) | -6.96 |  | -0.0070** | (0.0010) | -4.87 |  | -0.0025** | (0.0003) | -1.88 |  |
| 4th | 0.0087** | (0.0015) | 7.72 |  | 0.0052** | (0.0006) | 3.61 |  | 0.0078** | (0.0006) | 5.93 |  |
| Highest | 0.0322** | (0.0050) | 28.70 |  | 0.0254** | (0.0037) | 17.66 |  | 0.0233** | (0.0016) | 17.71 |  |
|  |  |  |  |  |  |  |  |  |  |  |  |  |
| Female household head | -0.0006 | (0.0007) | -0.57 | **-0.57** | 0.0002 | (0.0005) | 0.15 | **0.15** | -0.0002 | (0.0002) | -0.15 | **-0.15** |
|  |  |  |  |  |  |  |  |  |  |  |  |  |
| Education of household head |  |  |  | **30.72** |  |  |  | **13.95** |  |  |  | **10.19** |
| No education | 0.0175** | (0.0046) | 15.57 |  | 0.0120** | (0.0026) | 8.36 |  | 0.0051** | (0.0008) | 3.86 |  |
| Below secondary | -0.0012 | (0.0011) | -1.05 |  | 0.0004 | (0.0008) | 0.30 |  | 0.0003 | (0.0002) | 0.25 |  |
| Secondary or above | 0.0182** | (0.0054) | 16.20 |  | 0.0076** | (0.0029) | 5.29 |  | 0.0080** | (0.0015) | 6.08 |  |
|  |  |  |  |  |  |  |  |  |  |  |  |  |
| Household size |  |  |  | **-1.88** |  |  |  | **0.31** |  |  |  | **-1.48** |
| 1-2 members | 0.0015* | (0.0006) | 1.29 |  | 0.0003** | (0.0001) | 0.18 |  | 0.0001** | (0.0000) | 0.07 |  |
| 3-4 members | 0.0005 | (0.0014) | 0.42 |  | 0.0020* | (0.0008) | 1.37 |  | 0.0005 | (0.0003) | 0.39 |  |
| 5 or more members | -0.0040** | (0.0012) | -3.59 |  | -0.0018* | (0.0009) | -1.24 |  | -0.0026** | (0.0003) | -1.94 |  |
|  |  |  |  |  |  |  |  |  |  |  |  |  |
| Number of earners | 0.0003 | (0.0014) | 0.29 | **0.29** | 0.0022* | (0.0009) | 1.54 | **1.54** | 0.0017 | (0.0009) | 1.27 | **1.27** |
|  |  |  |  |  |  |  |  |  |  |  |  |  |
| Presence of elderly household member(s) | -0.0025 | (0.0017) | -2.22 | **-2.22** | -0.0001 | (0.0010) | -0.03 | **-0.03** | 0.0014* | (0.0006) | 1.04 | **1.04** |
|  |  |  |  |  |  |  |  |  |  |  |  |  |
| Presence of children under five years | 0.0021* | (0.0011) | 1.88 | **1.88** | 0.0015** | (0.0004) | 1.06 | **1.06** | -0.0002 | (0.0001) | -0.16 | **-0.16** |
|  |  |  |  |  |  |  |  |  |  |  |  |  |
| Presence of household member(s) with chronic illness | 0.0000 | (0.0003) | 0.01 | **0.01** | 0.0006 | (0.0004) | 0.41 | **0.41** | 0.0120** | (0.0008) | 9.14 | **9.14** |
|  |  |  |  |  |  |  |  |  |  |  |  |  |
| Source of healthcare |  |  |  | **-23.50** |  |  |  | **-7.34** |  |  |  | **-12.90** |
| Public only | 0.0015 | (0.0013) | 1.32 |  | 0.0019* | (0.0009) | 1.34 |  | 0.0005** | (0.0001) | 0.41 |  |
| Private only | 0.0068* | (0.0030) | 6.08 |  | 0.0018 | (0.0011) | 1.24 |  | -0.0009 | (0.0010) | -0.71 |  |
| Informal only | -0.0327** | (0.0047) | -29.17 |  | -0.0141** | (0.0018) | -9.78 |  | -0.0157** | (0.0009) | -11.90 |  |
| Public & private | -0.0010 | (0.0012) | -0.92 |  | -0.0002 | (0.0001) | -0.16 |  | -0.0011** | (0.0002) | -0.80 |  |
| Public & informal | -0.0002 | (0.0005) | -0.19 |  | 0.0000 | (0.0001) | 0.00 |  | -0.0003 | (0.0003) | -0.26 |  |
| Private & informal | -0.0002 | (0.0002) | -0.17 |  | 0.0001 | (0.0001) | 0.05 |  | 0.0003** | (0.0001) | 0.24 |  |
| Public, private & informal | -0.0005 | (0.0002) | -0.45 |  | 0.0000* | (0.0000) | -0.03 |  | 0.0002** | (0.0001) | 0.12 |  |
|  |  |  |  |  |  |  |  |  |  |  |  |  |
| Hospitalization of household members | -0.0046** | (0.0008) | -4.13 | **-4.13** | -0.0010* | (0.0001) | -0.66 | **-0.66** | -0.0029** | (0.0002) | -2.20 | **-0.66** |
|  |  |  |  |  |  |  |  |  |  |  |  |  |
|  |  |  |  |  |  |  |  |  |  |  |  |  |
| Characteristics | Panel C: Detailed decomposition: Difference due to coefficients | | | | | | | | | | | |
|  | 2005 | | | | 2010 | | | | 2016 | | | |
|  | Coefficient | Std. Err. | Percent | | Coefficient | Std. Err. | Percent | | Coefficient | Std. Err. | Percent | |
|  |  |  |  |  |  |  |  |  |  |  |  |  |
| Consumption expenditure quintile |  |  |  | **5.13** |  |  |  | **1.96** |  |  |  | **4.68** |
| Lowest | -0.0027* | (0.0012) | -2.37 |  | -0.0023 | (0.0014) | -1.59 |  | -0.0030** | (0.0011) | -2.27 |  |
| 2nd | 0.0027 | (0.0018) | 2.44 |  | 0.0018 | (0.0021) | 1.28 |  | 0.0005 | (0.0016) | 0.38 |  |
| 3rd | -0.0022 | (0.0028) | -1.96 |  | 0.0030 | (0.0025) | 2.05 |  | 0.0036 | (0.0024) | 2.76 |  |
| 4th | 0.0054 | (0.0044) | 4.81 |  | -0.0053 | (0.0042) | -3.67 |  | -0.0015 | (0.0038) | -1.13 |  |
| Highest | 0.0025 | (0.0064) | 2.21 |  | 0.0056 | (0.0067) | 3.89 |  | 0.0065 | (0.0050) | 4.94 |  |
|  |  |  |  |  |  |  |  |  |  |  |  |  |
| Female household head | -0.0019 | (0.0025) | -1.68 | **-1.68** | -0.0022 | (0.0026) | -1.55 | **-1.55** | 0.0002 | (0.0021) | 0.18 | **0.18** |
|  |  |  |  |  |  |  |  |  |  |  |  |  |
| Education of household head |  |  |  | **0.14** |  |  |  | **-2.29** |  |  |  | **-0.64** |
| No education | -0.0030 | (0.0044) | -2.69 |  | -0.0047 | (0.0047) | -3.27 |  | 0.0035 | (0.0037) | 2.68 |  |
| Below secondary | 0.0045 | (0.0046) | 3.99 |  | -0.0087 | (0.0045) | -6.01 |  | -0.0038 | (0.0044) | -2.88 |  |
| Secondary or above | -0.0013 | (0.0050) | -1.16 |  | 0.0101* | (0.0045) | 6.99 |  | -0.0006 | (0.0040) | -0.44 |  |
|  |  |  |  |  |  |  |  |  |  |  |  |  |
| Household size |  |  |  | **-0.99** |  |  |  | **4.02** |  |  |  | **1.86** |
| 1-2 members | 0.0000 | (0.0012) | 0.01 |  | -0.0012 | (0.0014) | -0.82 |  | 0.0006 | (0.0015) | 0.45 |  |
| 3-4 members | 0.0043 | (0.0069) | 3.84 |  | 0.0040 | (0.0059) | 2.75 |  | 0.0111* | (0.0052) | 8.41 |  |
| 5 or more members | -0.0054 | (0.0090) | -4.84 |  | 0.0030 | (0.0061) | 2.09 |  | -0.0092* | (0.0042) | -7.00 |  |
|  |  |  |  |  |  |  |  |  |  |  |  |  |
| Number of earners | 0.0071 | (0.0152) | 6.35 | **6.35** | -0.0094 | (0.0140) | -6.53 | **-6.53** | 0.0150 | (0.0137) | 11.43 | **11.43** |
|  |  |  |  |  |  |  |  |  |  |  |  |  |
| Presence of elderly household member(s) | -0.0003 | (0.0047) | -0.30 | **-0.30** | 0.0012 | (0.0042) | 0.81 | **0.81** | 0.0009 | (0.0040) | 0.68 | **0.68** |
|  |  |  |  |  |  |  |  |  |  |  |  |  |
| Presence of children under five years | 0.0104 | (0.0083) | 9.27 | **9.27** | 0.0126 | (0.0077) | 8.77 | **8.77** | 0.0066 | (0.0063) | 5.01 | **5.01** |
|  |  |  |  |  |  |  |  |  |  |  |  |  |
| Presence of household member(s) with chronic illness | 0.0118 | (0.0080) | 10.50 | **10.50** | 0.0135 | (0.0088) | 9.34 | **9.34** | -0.0023 | (0.0081) | -1.71 | **-1.71** |
|  |  |  |  |  |  |  |  |  |  |  |  |  |
| Source of healthcare |  |  |  | **-13.49** |  |  |  | **-10.49** |  |  |  | **4.03** |
| Public only | -0.0041 | (0.0027) | -3.62 |  | -0.0032 | (0.0028) | -2.24 |  | 0.0008 | (0.0020) | 0.61 |  |
| Private only | -0.0168 | (0.0087) | -15.02 |  | -0.0142 | (0.0076) | -9.88 |  | 0.0060 | (0.0044) | 4.56 |  |
| Informal only | 0.0023 | (0.0068) | 2.02 |  | 0.0027 | (0.0066) | 1.88 |  | 0.0004 | (0.0060) | 0.30 |  |
| Public & private | -0.0007 | (0.0008) | -0.61 |  | 0.0002 | (0.0009) | 0.17 |  | 0.0001 | (0.0010) | 0.09 |  |
| Public & informal | 0.0003 | (0.0005) | 0.25 |  | 0.0012 | (0.0008) | 0.86 |  | 0.0005 | (0.0010) | 0.36 |  |
| Private & informal | 0.0037 | (0.0023) | 3.27 |  | -0.0019 | (0.0013) | -1.30 |  | -0.0024 | (0.0018) | -1.86 |  |
| Public, private & informal | 0.0002 | (0.0004) | 0.22 |  | 0.0000 | (0.0002) | 0.02 |  | 0.0000 | (0.0003) | -0.03 |  |
|  |  |  |  |  |  |  |  |  |  |  |  |  |
| Hospitalization of household members | -0.0007 | (0.0015) | -0.64 | **-0.64** | -0.0005 | (0.0014) | -0.33 | **-0.33** | -0.0007 | (0.0022) | -0.50 | **-0.50** |
|  |  |  |  |  |  |  |  |  |  |  |  |  |
| Constant | 0.0056 | (0.0269) | 4.97 | **4.97** | 0.0540* | (0.0263) | 37.50 | **37.50** | 0.0204 | (0.0231) | 15.53 | **15.53** |
|  |  |  |  |  |  |  |  |  |  |  |  |  |

Std. Err. = standard error; * *p* ≤ 0.05, ** *p* ≤ 0.01
