# Supplementary material for: Rural‒urban disparities in household catastrophic health expenditure in Bangladesh: a multivariate decomposition analysis
Source: Int J Equity Health. 2024 Feb 27;23:43. doi: 10.1186/s12939-024-02125-3 (PMC10898052; doi:10.1186/s12939-024-02125-3)
Supplement: Supplementary file 4 — Additional file 4. Mean CHE incidence among rural and urban households; aggregate and detailed decomposition of the difference in CHE incidence between rural and urban areas: normative food expenditure method, 40% threshold. [file 12939_2024_2125_MOESM4_ESM.docx]

**Additional Table 4**: Rural-urban differences in catastrophic health expenditure (CHE) incidence, normative food expenditure method, 40% threshold

|  | Panel A: Aggregate decomposition | | | | | | | | | | | |
| --- | --- | --- | --- | --- | --- | --- | --- | --- | --- | --- | --- | --- |
|  | 2005 | | | | 2010 | | | | 2016 | | | |
|  | Coefficient | Std. Err. | Percent | | Coefficient | Std. Err. | Percent | | Coefficient | Std. Err. | Percent | |
| CHE incidence |  |  |  |  |  |  |  |  |  |  |  |  |
| Rural | 0.1649 | (0.0075) |  |  | 0.1624 | (0.0070) |  |  | 0.2184 | (0.0057) |  |  |
| Urban | 0.1181 | (0.0109) |  |  | 0.0880 | (0.0080) |  |  | 0.1303 | (0.0078) |  |  |
|  |  |  |  |  |  |  |  |  |  |  |  |  |
| Total difference | 0.0468** | (0.0125) |  | | 0.0744** | (0.0090) |  | | 0.0881** | (0.0069) |  | |
|  |  |  |  | |  |  |  | |  |  |  | |
| Difference due to characteristics | 0.0291** | (0.0062) | 62.14 | | 0.0249** | (0.0037) | 33.47 | | 0.0408** | (0.0027) | 46.29 | |
| Difference due to coefficients | 0.0177 | (0.0133) | 37.86 | | 0.0495** | (0.0093) | 66.53 | | 0.0473** | (0.0072) | 53.71 | |
|  |  |  |  |  |  |  |  |  |  |  |  |  |
|  | Panel B: Detailed decomposition: Difference due to characteristics | | | | | | | | | | | |
| Characteristics | 2005 | | | | 2010 | | | | 2016 | | | |
|  | Coefficient | Std. Err. | Percent | | Coefficient | Std. Err. | Percent | | Coefficient | Std. Err. | Percent | |
| Consumption expenditure quintile |  |  |  | **69.04** |  |  |  | **28.49** |  |  |  | **40.65** |
| Lowest | 0.0123** | (0.0023) | 26.30 |  | 0.0124** | (0.0015) | 16.62 |  | 0.0173* | (0.0009) | 19.58 |  |
| 2nd | 0.0053** | (0.0014) | 11.28 |  | 0.0027** | (0.0009) | 3.57 |  | 0.0026** | (0.0005) | 2.90 |  |
| 3rd | -0.0022* | (0.0010) | -4.69 |  | -0.0017** | (0.0005) | -2.25 |  | -0.0008** | (0.0002) | -0.85 |  |
| 4th | 0.0039** | (0.0012) | 8.42 |  | 0.0026** | (0.0004) | 3.50 |  | 0.0039** | (0.0004) | 4.37 |  |
| Highest | 0.0130** | (0.0036) | 27.73 |  | 0.0052* | (0.0025) | 7.05 |  | 0.0129** | (0.0013) | 14.65 |  |
|  |  |  |  |  |  |  |  |  |  |  |  |  |
| Female household head | -0.0005 | (0.0005) | -1.01 | **-1.01** | -0.0003 | (0.0004) | -0.42 | **-0.42** | -0.0005** | (0.0001) | -0.60 | **-0.60** |
|  |  |  |  |  |  |  |  |  |  |  |  |  |
| Education of household head |  |  |  | **43.99** |  |  |  | **17.05** |  |  |  | **13.41** |
| No education | 0.0101** | (0.0033) | 21.62 |  | 0.0067** | (0.0020) | 8.95 |  | 0.0039** | (0.0007) | 4.46 |  |
| Below secondary | -0.0009 | (0.0008) | -1.96 |  | -0.0007 | (0.0006) | -0.96 |  | 0.0005** | (0.0002) | 0.59 |  |
| Secondary or above | 0.0114** | (0.0039) | 24.33 |  | 0.0067** | (0.0021) | 9.06 |  | 0.0074** | (0.0013) | 8.36 |  |
|  |  |  |  |  |  |  |  |  |  |  |  |  |
| Household size |  |  |  | **-2.21** |  |  |  | **-1.06** |  |  |  | **-2.67** |
| 1-2 members | 0.0013** | (0.0004) | 2.74 |  | 0.0001 | (0.0001) | 0.13 |  | 0.0001** | (0.0000) | 0.10 |  |
| 3-4 members | 0.0009 | (0.0010) | 1.89 |  | 0.0002 | (0.0006) | 0.32 |  | 0.0002 | (0.0003) | 0.28 |  |
| 5 or more members | -0.0032** | (0.0009) | -6.84 |  | -0.0011 | (0.0006) | -1.51 |  | -0.0027** | (0.0003) | -3.05 |  |
|  |  |  |  |  |  |  |  |  |  |  |  |  |
| Number of earners | 0.0008 | (0.0010) | 1.61 | **1.61** | 0.0016* | (0.0007) | 2.21 | **2.21** | 0.0027** | (0.0008) | 3.04 | **3.04** |
|  |  |  |  |  |  |  |  |  |  |  |  |  |
| Presence of elderly household member(s) | -0.0020 | (0.0012) | -4.26 | **-4.26** | -0.0008 | (0.0008) | -1.07 | **-1.07** | 0.0012* | (0.0005) | 1.35 | **1.35** |
|  |  |  |  |  |  |  |  |  |  |  |  |  |
| Presence of children under five years | 0.0017* | (0.0008) | 3.64 | **3.64** | 0.0005 | (0.0003) | 0.68 | **0.68** | -0.0001 | (0.0001) | -0.15 | **-0.15** |
|  |  |  |  |  |  |  |  |  |  |  |  |  |
| Presence of household member(s) with chronic illness | 0.0002 | (0.0002) | 0.45 | **0.45** | 0.0004 | (0.0003) | 0.53 | **0.53** | 0.0097** | (0.0007) | 11.05 | **11.05** |
|  |  |  |  |  |  |  |  |  |  |  |  |  |
| Source of healthcare |  |  |  | **-41.66** |  |  |  | **-12.08** |  |  |  | **-17.01** |
| Public only | 0.0015 | (0.0009) | 3.25 |  | 0.0013* | (0.0006) | 1.81 |  | 0.0005** | (0.0001) | 0.56 |  |
| Private only | 0.0050** | (0.0019) | 10.61 |  | 0.0012 | (0.0007) | 1.56 |  | -0.0012 | (0.0008) | -1.31 |  |
| Informal only | -0.0242** | (0.0037) | -51.68 |  | -0.0114** | (0.0013) | -15.32 |  | -0.0137* | (0.0007) | -15.57 |  |
| Public & private | -0.0014* | (0.0007) | -2.99 |  | -0.0002* | (0.0001) | -0.26 |  | -0.0009** | (0.0002) | -0.98 |  |
| Public & informal | 0.0000 | (0.0003) | -0.09 |  | 0.0001 | (0.0001) | 0.12 |  | -0.0001 | (0.0003) | -0.13 |  |
| Private & informal | 0.0000 | (0.0001) | -0.04 |  | 0.0000 | (0.0001) | 0.04 |  | 0.0002** | (0.0001) | 0.27 |  |
| Public, private & informal | -0.0003* | (0.0002) | -0.72 |  | 0.0000 | (0.0000) | -0.03 |  | 0.0001** | (0.0000) | 0.15 |  |
|  |  |  |  |  |  |  |  |  |  |  |  |  |
| Hospitalization of household members | -0.0035** | (0.0006) | -7.48 | **-7.48** | -0.0006** | (0.0001) | -0.86 | **-0.86** | -0.0024** | (0.0002) | -2.77 | **-0.86** |
|  |  |  |  |  |  |  |  |  |  |  |  |  |
|  |  |  |  |  |  |  |  |  |  |  |  |  |
| Characteristics | Panel C: Detailed decomposition:Difference due to coefficients | | | | | | | | | | | |
|  | 2005 | | | | 2010 | | | | 2016 | | | |
|  | Coefficient | Std. Err. | Percent | | Coefficient | Std. Err. | Percent | | Coefficient | Std. Err. | Percent | |
|  |  |  |  |  |  |  |  |  |  |  |  |  |
| Consumption expenditure quintile |  |  |  | **16.13** |  |  |  | **10.99** |  |  |  | **8.12** |
| Lowest | -0.0022 | (0.0014) | -4.75 |  | -0.0032** | (0.0011) | -4.26 |  | -0.0038** | (0.0008) | -4.30 |  |
| 2nd | 0.0004 | (0.0020) | 0.87 |  | 0.0004 | (0.0017) | 0.60 |  | -0.0012 | (0.0015) | -1.34 |  |
| 3rd | -0.0014 | (0.0028) | -2.97 |  | 0.0036 | (0.0026) | 4.78 |  | 0.0090* | (0.0022) | 10.16 |  |
| 4th | 0.0092 | (0.0053) | 19.66 |  | -0.0043 | (0.0034) | -5.80 |  | 0.0009 | (0.0033) | 0.99 |  |
| Highest | 0.0016 | (0.0071) | 3.32 |  | 0.0117* | (0.0050) | 15.67 |  | 0.0023 | (0.0046) | 2.61 |  |
|  |  |  |  |  |  |  |  |  |  |  |  |  |
| Female household head | -0.0003 | (0.0031) | -0.61 | **-0.61** | -0.0031 | (0.0021) | -4.18 | **-4.18** | -0.0001 | (0.0021) | -0.11 | **-0.11** |
|  |  |  |  |  |  |  |  |  |  |  |  |  |
| Education of household head |  |  |  | **0.07** |  |  |  | **-2.34** |  |  |  | **-0.04** |
| No education | 0.0006 | (0.0052) | 1.35 |  | -0.0030 | (0.0039) | -4.02 |  | -0.0012 | (0.0033) | -1.33 |  |
| Below secondary | -0.0002 | (0.0047) | -0.41 |  | -0.0041 | (0.0037) | -5.55 |  | 0.0005 | (0.0041) | 0.58 |  |
| Secondary or above | -0.0004 | (0.0057) | -0.87 |  | 0.0054 | (0.0039) | 7.23 |  | 0.0006 | (0.0038) | 0.71 |  |
|  |  |  |  |  |  |  |  |  |  |  |  |  |
| Household size |  |  |  | **-17.64** |  |  |  | **7.50** |  |  |  | **3.33** |
| 1-2 members | 0.0007 | (0.0013) | 1.47 |  | -0.0011 | (0.0011) | -1.49 |  | 0.0003 | (0.0014) | 0.35 |  |
| 3-4 members | 0.0018 | (0.0074) | 3.75 |  | 0.0073 | (0.0050) | 9.75 |  | 0.0106* | (0.0048) | 12.01 |  |
| 5 or more members | -0.0107 | (0.0105) | -22.86 |  | -0.0006 | (0.0050) | -0.76 |  | -0.0080 | (0.0041) | -9.03 |  |
|  |  |  |  |  |  |  |  |  |  |  |  |  |
| Number of earners | 0.0039 | (0.0175) | 8.35 | **8.35** | -0.0043 | (0.0127) | -5.81 | **-5.81** | 0.0052 | (0.0131) | 5.87 | **5.87** |
|  |  |  |  |  |  |  |  |  |  |  |  |  |
| Presence of elderly household member(s) | -0.0007 | (0.0051) | -1.40 | **-1.40** | 0.0038 | (0.0036) | 5.04 | **5.04** | -0.0024 | (0.0034) | -2.77 | **-2.77** |
|  |  |  |  |  |  |  |  |  |  |  |  |  |
| Presence of children under five years | 0.0193 | (0.0110) | 41.20 | **41.20** | 0.0108 | (0.0062) | 14.56 | **14.56** | 0.0045 | (0.0055) | 5.14 | **5.14** |
|  |  |  |  |  |  |  |  |  |  |  |  |  |
| Presence of household member(s) with chronic illness | 0.0132 | (0.0097) | 28.26 | **28.26** | 0.0130 | (0.0073) | 17.50 | **17.50** | 0.0041 | (0.0074) | 4.60 | **4.60** |
|  |  |  |  |  |  |  |  |  |  |  |  |  |
| Source of healthcare |  |  |  | **-49.08** |  |  |  | **-26.67** |  |  |  | **4.18** |
| Public only | -0.0061 | (0.0034) | -13.03 |  | -0.0020 | (0.0025) | -2.71 |  | 0.0008 | (0.0018) | 0.88 |  |
| Private only | -0.0189 | (0.0101) | -40.49 |  | -0.0179** | (0.0067) | -24.06 |  | 0.0042 | (0.0039) | 4.77 |  |
| Informal only | -0.0018 | (0.0076) | -3.83 |  | -0.0001 | (0.0065) | -0.08 |  | 0.0004 | (0.0052) | 0.42 |  |
| Public & private | 0.0000 | (0.0009) | 0.02 |  | 0.0001 | (0.0008) | 0.18 |  | 0.0002 | (0.0009) | 0.19 |  |
| Public & informal | 0.0001 | (0.0006) | 0.17 |  | 0.0021* | (0.0010) | 2.85 |  | 0.0010 | (0.0009) | 1.08 |  |
| Private & informal | 0.0034 | (0.0025) | 7.32 |  | -0.0020 | (0.0012) | -2.71 |  | -0.0027 | (0.0016) | -3.09 |  |
| Public, private & informal | 0.0004 | (0.0004) | 0.76 |  | -0.0001 | (0.0002) | -0.14 |  | -0.0001 | (0.0002) | -0.07 |  |
|  |  |  |  |  |  |  |  |  |  |  |  |  |
| Hospitalization of household members | 0.0003 | (0.0017) | 0.62 | **0.62** | -0.0007 | (0.0011) | -0.95 | **-0.95** | -0.0023 | (0.0019) | -2.60 | **-2.60** |
|  |  |  |  |  |  |  |  |  |  |  |  |  |
| Constant | 0.0056 | (0.0295) | 11.96 | **11.96** | 0.0379 | (0.0245) | 50.90 | **50.90** | 0.0247 | (0.0213) | 28.00 | **28.00** |
|  |  |  |  |  |  |  |  |  |  |  |  |  |

Std. Err. = standard error; * *p* ≤ 0.05, ** *p* ≤ 0.01
