# Supplementary material for: Rural‒urban disparities in household catastrophic health expenditure in Bangladesh: a multivariate decomposition analysis
Source: Int J Equity Health. 2024 Feb 27;23:43. doi: 10.1186/s12939-024-02125-3 (PMC10898052; doi:10.1186/s12939-024-02125-3)
Supplement: Supplementary file 5 — Additional file 5. Mean CHE incidence among rural and urban households; aggregate and detailed decomposition of the difference in CHE incidence between rural and urban households: budget share method, 10% threshold. [file 12939_2024_2125_MOESM5_ESM.docx]

**Additional Table 5**: Rural-urban differences in catastrophic health expenditure (CHE) incidence, budget share method, 10% threshold

|  | Panel A: Aggregate decomposition | | | | | | | | | | | |
| --- | --- | --- | --- | --- | --- | --- | --- | --- | --- | --- | --- | --- |
|  | 2005 | | | | 2010 | | | | 2016 | | | |
|  | Coefficient | Std. Err. | Percent | | Coefficient | Std. Err. | Percent | | Coefficient | Std. Err. | Percent | |
| CHE incidence |  |  |  |  |  |  |  |  |  |  |  |  |
| Rural | 0.2392 | (0.0086) |  |  | 0.2478 | (0.0090) |  |  | 0.3881 | (0.0069) |  |  |
| Urban | 0.2346 | (0.0153) |  |  | 0.2036 | (0.0131) |  |  | 0.3287 | (0.0134) |  |  |
|  |  |  |  |  |  |  |  |  |  |  |  |  |
| Total difference | 0.0046 | (0.0163) | 100.00 | | 0.0442** | (0.0130) | 100.00 | | 0.0594** | (0.0099) | 100.00 | |
|  |  |  |  | |  |  |  | |  |  |  | |
| Difference due to characteristics | -0.0253** | (0.0084) | -550.17 | | -0.0134** | (0.0051) | -30.31 | | -0.0032 | (0.0037) | -5.39 | |
| Difference due to coefficients | 0.0299 | (0.0186) | 650.17 | | 0.0575** | (0.0140) | 130.31 | | 0.0626** | (0.0107) | 105.39 | |
|  |  |  |  |  |  |  |  |  |  |  |  |  |
|  | Panel B: Detailed decomposition: Difference due to characteristics | | | | | | | | | | | |
| Characteristics | 2005 | | | | 2010 | | | | 2016 | | | |
|  | Coefficient | Std. Err. | Percent | | Coefficient | Std. Err. | Percent | | Coefficient | Std. Err. | Percent | |
| Consumption expenditure quintile |  |  |  | **-394.10** |  |  |  | **-37.59** |  |  |  | **-2.07** |
| Lowest | -0.0083** | (0.0022) | -181.27 |  | -0.0041** | (0.0015) | -9.21 |  | -0.0032* | (0.0014) | -5.37 |  |
| 2nd | -0.0016 | (0.0017) | -34.56 |  | -0.0035** | (0.0011) | -7.98 |  | -0.0001 | (0.0009) | -0.09 |  |
| 3rd | 0.0015 | (0.0009) | 33.22 |  | 0.0003 | (0.0004) | 0.73 |  | 0.0007 | (0.0005) | 1.18 |  |
| 4th | -0.0015 | (0.0010) | -33.56 |  | -0.0011** | (0.0003) | -2.37 |  | -0.0006 | (0.0006) | -1.07 |  |
| Highest | -0.0082* | (0.0036) | -177.93 |  | -0.0083** | (0.0027) | -18.76 |  | 0.0020 | (0.0029) | 3.28 |  |
|  |  |  |  |  |  |  |  |  |  |  |  |  |
| Female household head | -0.0006 | (0.0006) | -14.00 | **-14.00** | 0.0002 | (0.0004) | 0.39 | **0.39** | -0.0001 | (0.0002) | -0.09 | **-0.09** |
|  |  |  |  |  |  |  |  |  |  |  |  |  |
| Education of household head |  |  |  | **590.50** |  |  |  | **30.77** |  |  |  | **20.67** |
| No education | 0.0137** | (0.0034) | 297.19 |  | 0.0072** | (0.0018) | 16.40 |  | 0.0048 | (0.0027) | 8.14 |  |
| Below secondary | -0.0010 | (0.0008) | -21.66 |  | -0.0007 | (0.0006) | -1.49 |  | 0.0002 | (0.0004) | 0.38 |  |
| Secondary or above | 0.0145** | (0.0042) | 314.97 |  | 0.0070** | (0.0019) | 15.86 |  | 0.0072 | (0.0045) | 12.15 |  |
|  |  |  |  |  |  |  |  |  |  |  |  |  |
| Household size |  |  |  | **-25.95** |  |  |  | **-3.07** |  |  |  | **-4.66** |
| 1-2 members | 0.0015** | (0.0005) | 33.60 |  | 0.0003** | (0.0001) | 0.57 |  | 0.0002* | (0.0001) | 0.28 |  |
| 3-4 members | 0.0011 | (0.0011) | 23.90 |  | 0.0010 | (0.0006) | 2.25 |  | 0.0013 | (0.0007) | 2.13 |  |
| 5 or more members | -0.0038** | (0.0009) | -83.45 |  | -0.0026** | (0.0007) | -5.89 |  | -0.0042* | (0.0017) | -7.07 |  |
|  |  |  |  |  |  |  |  |  |  |  |  |  |
| Number of earners | 0.0026* | (0.0011) | 55.59 | **55.59** | 0.0028** | (0.0008) | 6.44 | **6.44** | 0.0030 | (0.0021) | 5.07 | **5.07** |
|  |  |  |  |  |  |  |  |  |  |  |  |  |
| Presence of elderly household member(s) | -0.0006 | (0.0013) | -13.48 | **-13.48** | 0.0010 | (0.0008) | 2.26 | **2.26** | 0.0025 | (0.0015) | 4.22 | **4.22** |
|  |  |  |  |  |  |  |  |  |  |  |  |  |
| Presence of children under five years | 0.0010 | (0.0008) | 21.37 | **21.37** | 0.0008** | (0.0003) | 1.90 | **1.90** | 0.0000 | (0.0002) | 0.00 | **0.00** |
|  |  |  |  |  |  |  |  |  |  |  |  |  |
| Presence of household member(s) with chronic illness | 0.0004 | (0.0002) | 8.20 | **8.20** | 0.0007** | (0.0002) | 1.56 | **1.56** | 0.0248* | (0.0110) | 41.68 | **41.68** |
|  |  |  |  |  |  |  |  |  |  |  |  |  |
| Source of healthcare |  |  |  | **-681.79** |  |  |  | **-31.44** |  |  |  | **-61.41** |
| Public only | -0.0002 | (0.0010) | -4.77 |  | 0.0028** | (0.0009) | 6.27 |  | 0.0012* | (0.0006) | 2.05 |  |
| Private only | 0.0031 | (0.0025) | 67.54 |  | 0.0035** | (0.0013) | 7.92 |  | -0.0037 | (0.0023) | -6.17 |  |
| Informal only | -0.0333** | (0.0039) | -724.04 |  | -0.0197** | (0.0024) | -44.68 |  | -0.0325* | (0.0141) | -54.70 |  |
| Public & private | -0.0002 | (0.0011) | -4.68 |  | -0.0002 | (0.0001) | -0.50 |  | -0.0019* | (0.0009) | -3.11 |  |
| Public & informal | -0.0001 | (0.0004) | -1.11 |  | -0.0001 | (0.0001) | -0.23 |  | -0.0007 | (0.0006) | -1.13 |  |
| Private & informal | -0.0003** | (0.0001) | -7.26 |  | 0.0000 | (0.0001) | -0.03 |  | 0.0006* | (0.0003) | 1.05 |  |
| Public, private & informal | -0.0003 | (0.0002) | -7.47 |  | -0.0001** | (0.0000) | -0.19 |  | 0.0004 | (0.0002) | 0.60 |  |
|  |  |  |  |  |  |  |  |  |  |  |  |  |
| Hospitalization of household members | -0.0044** | (0.0008) | -96.52 | **-96.52** | -0.0007** | (0.0001) | -1.50 | **-1.50** | -0.0052* | (0.0023) | -8.83 | **-1.50** |
|  |  |  |  |  |  |  |  |  |  |  |  |  |
|  |  |  |  |  |  |  |  |  |  |  |  |  |
| Characteristics | Panel C: Detailed decomposition:Difference due to coefficients | | | | | | | | | | | |
|  | 2005 | | | | 2010 | | | | 2016 | | | |
|  | Coefficient | Std. Err. | Percent | | Coefficient | Std. Err. | Percent | | Coefficient | Std. Err. | Percent | |
|  |  |  |  |  |  |  |  |  |  |  |  |  |
| Consumption expenditure quintile |  |  |  | **203.36** |  |  |  | **6.04** |  |  |  | **8.58** |
| Lowest | -0.0031 | (0.0030) | -66.50 |  | 0.0000 | (0.0025) | -0.05 |  | -0.0039* | (0.0017) | -6.57 |  |
| 2nd | 0.0011 | (0.0034) | 22.92 |  | 0.0028 | (0.0035) | 6.31 |  | 0.0014 | (0.0028) | 2.36 |  |
| 3rd | 0.0017 | (0.0043) | 36.94 |  | -0.0040 | (0.0041) | -9.08 |  | 0.0083* | (0.0038) | 14 |  |
| 4th | 0.0002 | (0.0069) | 4.14 |  | -0.0050 | (0.0053) | -11.23 |  | -0.0016 | (0.0045) | -2.72 |  |
| Highest | 0.0095 | (0.0114) | 205.86 |  | 0.0089 | (0.0085) | 20.09 |  | 0.0009 | (0.0068) | 1.51 |  |
|  |  |  |  |  |  |  |  |  |  |  |  |  |
| Female household head | -0.0029 | (0.0038) | -63.43 | **-63.43** | -0.0045 | (0.0038) | -10.14 | **-10.14** | 0.0047 | (0.0031) | 7.97 | **7.97** |
|  |  |  |  |  |  |  |  |  |  |  |  |  |
| Education of household head |  |  |  | **2.74** |  |  |  | **-2.33** |  |  |  | **-1.37** |
| No education | -0.0019 | (0.0074) | -41.32 |  | 0.0034 | (0.0065) | 7.71 |  | 0.0016 | (0.0052) | 2.67 |  |
| Below secondary | 0.0030 | (0.0067) | 66.18 |  | -0.0068 | (0.0062) | -15.40 |  | -0.0027 | (0.0059) | -4.6 |  |
| Secondary or above | -0.0010 | (0.0077) | -22.12 |  | 0.0024 | (0.0057) | 5.36 |  | 0.0003 | (0.0050) | 0.56 |  |
|  |  |  |  |  |  |  |  |  |  |  |  |  |
| Household size |  |  |  | **-739.27** |  |  |  | **-8.83** |  |  |  | **-3.33** |
| 1-2 members | 0.0032 | (0.0021) | 69.46 |  | 0.0009 | (0.0021) | 1.93 |  | 0.0007 | (0.0022) | 1.23 |  |
| 3-4 members | -0.0113 | (0.0115) | -246.23 |  | 0.0023 | (0.0088) | 5.29 |  | -0.0007 | (0.0078) | -1.25 |  |
| 5 or more members | -0.0259 | (0.0171) | -562.50 |  | -0.0071 | (0.0088) | -16.05 |  | -0.0020 | (0.0056) | -3.31 |  |
|  |  |  |  |  |  |  |  |  |  |  |  |  |
| Number of earners | -0.0012 | (0.0250) | -25.57 | **-25.57** | -0.0159 | (0.0221) | -36.08 | **-36.08** | -0.0054 | (0.0281) | -9.16 | **-9.16** |
|  |  |  |  |  |  |  |  |  |  |  |  |  |
| Presence of elderly household member(s) | 0.0086 | (0.0074) | 186.94 | **186.94** | 0.0147* | (0.0060) | 33.36 | **33.36** | -0.0064 | (0.0054) | -10.81 | **-10.81** |
|  |  |  |  |  |  |  |  |  |  |  |  |  |
| Presence of children under five years | 0.0259 | (0.0142) | 563.75 | **563.75** | 0.0223* | (0.0105) | 50.46 | **50.46** | 0.0162 | (0.0091) | 27.33 | **27.33** |
|  |  |  |  |  |  |  |  |  |  |  |  |  |
| Presence of household member(s) with chronic illness | 0.0067 | (0.0141) | 146.45 | **146.45** | 0.0013 | (0.0131) | 2.94 | **2.94** | -0.0065 | (0.0105) | -10.97 | **-10.97** |
|  |  |  |  |  |  |  |  |  |  |  |  |  |
| Source of healthcare |  |  |  | **-625.99** |  |  |  | **-105.81** |  |  |  | **28.46** |
| Public only | -0.0020 | (0.0042) | -44.29 |  | -0.0056 | (0.0046) | -12.78 |  | -0.0009 | (0.0034) | -1.49 |  |
| Private only | -0.0301* | (0.0148) | -653.89 |  | -0.0319** | (0.0120) | -72.30 |  | 0.0086 | (0.0069) | 14.47 |  |
| Informal only | 0.0020 | (0.0128) | 42.41 |  | -0.0067 | (0.0138) | -15.25 |  | 0.0062 | (0.0100) | 10.5 |  |
| Public & private | -0.0025 | (0.0018) | -54.99 |  | -0.0001 | (0.0014) | -0.20 |  | -0.0013 | (0.0016) | -2.16 |  |
| Public & informal | 0.0008 | (0.0010) | 18.11 |  | 0.0017 | (0.0011) | 3.94 |  | 0.0026 | (0.0014) | 4.3 |  |
| Private & informal | 0.0024 | (0.0037) | 52.47 |  | -0.0045* | (0.0023) | -10.19 |  | 0.0024 | (0.0028) | 4.05 |  |
| Public, private & informal | 0.0007 | (0.0007) | 14.19 |  | 0.0004 | (0.0005) | 0.97 |  | -0.0007 | (0.0006) | -1.21 |  |
|  |  |  |  |  |  |  |  |  |  |  |  |  |
| Hospitalization of household members | -0.0009 | (0.0029) | -19.14 | **-19.14** | 0.0023 | (0.0019) | 5.24 | **5.24** | 0.0024 | (0.0045) | 4.11 | **4.11** |
|  |  |  |  |  |  |  |  |  |  |  |  |  |
| Constant | 0.0469 | (0.0492) | 1020.32 | **1020.32** | 0.0863* | (0.0427) | 195.47 | **195.47** | 0.0384 | (0.0353) | 64.59 | **64.59** |
|  |  |  |  |  |  |  |  |  |  |  |  |  |

Std. Err. = standard error; * *p* ≤ 0.05, ** *p* ≤ 0.01
