# Supplementary material for: Rural‒urban disparities in household catastrophic health expenditure in Bangladesh: a multivariate decomposition analysis
Source: Int J Equity Health. 2024 Feb 27;23:43. doi: 10.1186/s12939-024-02125-3 (PMC10898052; doi:10.1186/s12939-024-02125-3)
Supplement: Supplementary file 6 — Additional file 6. Mean CHE incidence among rural and urban households; aggregate and detailed decomposition of the difference in CHE incidence between rural and urban households: actual food expenditure method, 40% threshold. [file 12939_2024_2125_MOESM6_ESM.docx]

**Additional Table 6**: Rural-urban differences in catastrophic health expenditure (CHE) incidence, actual food expenditure method, 40% threshold

|  | Panel A: Aggregate decomposition | | | | | | | | | | | |
| --- | --- | --- | --- | --- | --- | --- | --- | --- | --- | --- | --- | --- |
|  | 2005 | | | | 2010 | | | | 2016 | | | |
|  | Coefficient | Std. Err. | Percent | | Coefficient | Std. Err. | Percent | | Coefficient | Std. Err. | Percent | |
| CHE incidence |  |  |  |  |  |  |  |  |  |  |  |  |
| Rural | 0.1181 | (0.0065) |  |  | 0.1220 | (0.0060) |  |  | 0.1719 | (0.0049) |  |  |
| Urban | 0.0991 | (0.0106) |  |  | 0.0719 | (0.0068) |  |  | 0.1162 | (0.0067) |  |  |
|  |  |  |  |  |  |  |  |  |  |  |  |  |
| Total difference | 0.0190 | (0.0115) | 100.00 | | 0.0501** | (0.0084) | 100.00 | | 0.0557** | (0.0067) | 100.00 | |
|  |  |  |  | |  |  |  | |  |  |  | |
| Difference due to characteristics | -0.0158* | (0.0068) | -82.82 | | -0.0040 | (0.0037) | -7.97 | | 0.0002 | (0.0028) | 0.26 | |
| Difference due to coefficients | 0.0348** | (0.0137) | 182.82 | | 0.0541** | (0.0092) | 107.97 | | 0.0556** | (0.0073) | 99.74 | |
|  |  |  |  |  |  |  |  |  |  |  |  |  |
|  | Panel B: Detailed decomposition: Difference due to characteristics | | | | | | | | | | | |
| Characteristics | 2005 | | | | 2010 | | | | 2016 | | | |
|  | Coefficient | Std. Err. | Percent | | Coefficient | Std. Err. | Percent | | Coefficient | Std. Err. | Percent | |
| Consumption expenditure quintile |  |  |  | **-49.07** |  |  |  | **-23.73** |  |  |  | **1.71** |
| Lowest | -0.0057** | (0.0016) | -30.07 |  | -0.0030* | (0.0012) | -6.04 |  | 0.0003 | (0.0060) | 0.49 |  |
| 2nd | 0.0004 | (0.0011) | 1.96 |  | -0.0014 | (0.0008) | -2.75 |  | 0.0002 | (0.0041) | 0.33 |  |
| 3rd | 0.0010 | (0.0006) | 5.14 |  | -0.0001 | (0.0003) | -0.14 |  | 0.0000 | (0.0008) | -0.06 |  |
| 4th | -0.0006 | (0.0007) | -3.04 |  | -0.0004 | (0.0002) | -0.71 |  | 0.0000 | (0.0004) | 0.03 |  |
| Highest | -0.0044 | (0.0025) | -23.06 |  | -0.0071* | (0.0029) | -14.09 |  | 0.0005 | (0.0113) | 0.92 |  |
|  |  |  |  |  |  |  |  |  |  |  |  |  |
| Female household head | -0.0006 | (0.0004) | -2.98 | **-2.98** | -0.0004 | (0.0003) | -0.69 | **-0.69** | 0.0001 | (0.0010) | 0.08 | **0.08** |
|  |  |  |  |  |  |  |  |  |  |  |  |  |
| Education of household head |  |  |  | **85.54** |  |  |  | **26.80** |  |  |  | **-3.36** |
| No education | 0.0079** | (0.0024) | 41.76 |  | 0.0077** | (0.0018) | 15.41 |  | -0.0007 | (0.0150) | -1.21 |  |
| Below secondary | -0.0008 | (0.0006) | -3.99 |  | -0.0001 | (0.0004) | -0.12 |  | -0.0001 | (0.0013) | -0.11 |  |
| Secondary or above | 0.0091** | (0.0032) | 47.77 |  | 0.0058** | (0.0017) | 11.51 |  | -0.0011 | (0.0253) | -2.04 |  |
|  |  |  |  |  |  |  |  |  |  |  |  |  |
| Household size |  |  |  | **-9.39** |  |  |  | **-1.57** |  |  |  | **0.40** |
| 1-2 members | 0.0011** | (0.0003) | 5.73 |  | 0.0002** | (0.0001) | 0.40 |  | 0.0000 | (0.0003) | -0.03 |  |
| 3-4 members | 0.0002 | (0.0007) | 1.24 |  | 0.0009* | (0.0004) | 1.87 |  | -0.0001 | (0.0028) | -0.23 |  |
| 5 or more members | -0.0031** | (0.0008) | -16.36 |  | -0.0019** | (0.0007) | -3.84 |  | 0.0004 | (0.0082) | 0.66 |  |
|  |  |  |  |  |  |  |  |  |  |  |  |  |
| Number of earners | 0.0009 | (0.0007) | 4.77 | **4.77** | 0.0017** | (0.0006) | 3.30 | **3.30** | -0.0003 | (0.0064) | -0.51 | **-0.51** |
|  |  |  |  |  |  |  |  |  |  |  |  |  |
| Presence of elderly household member(s) | -0.0007 | (0.0009) | -3.69 | **-3.69** | 0.0002 | (0.0005) | 0.36 | **0.36** | -0.0004 | (0.0077) | -0.62 | **-0.62** |
|  |  |  |  |  |  |  |  |  |  |  |  |  |
| Presence of children under five years | 0.0012* | (0.0006) | 6.38 | **6.38** | 0.0006* | (0.0003) | 1.18 | **1.18** | 0.0000 | (0.0003) | 0.03 | **0.03** |
|  |  |  |  |  |  |  |  |  |  |  |  |  |
| Presence of household member(s) with chronic illness | 0.0003 | (0.0001) | 1.31 | **1.31** | 0.0002 | (0.0001) | 0.33 | **0.33** | -0.0012 | (0.0273) | -2.21 | **-2.21** |
|  |  |  |  |  |  |  |  |  |  |  |  |  |
| Source of healthcare |  |  |  | **-102.68** |  |  |  | **-13.11** |  |  |  | **4.23** |
| Public only | 0.0004 | (0.0006) | 2.07 |  | 0.0006 | (0.0004) | 1.18 |  | -0.0001 | (0.0010) | -0.08 |  |
| Private only | 0.0022 | (0.0015) | 11.69 |  | 0.0011* | (0.0005) | 2.14 |  | 0.0002 | (0.0048) | 0.39 |  |
| Informal only | -0.0215** | (0.0034) | -112.87 |  | -0.0081** | (0.0022) | -16.16 |  | 0.0021 | (0.0456) | 3.69 |  |
| Public & private | -0.0005 | (0.0006) | -2.82 |  | -0.0001* | (0.0001) | -0.26 |  | 0.0001 | (0.0028) | 0.23 |  |
| Public & informal | 0.0002 | (0.0002) | 0.90 |  | 0.0000 | (0.0001) | -0.01 |  | 0.0001 | (0.0011) | 0.09 |  |
| Private & informal | -0.0001 | (0.0001) | -0.57 |  | 0.0000 | (0.0000) | 0.04 |  | 0.0000 | (0.0007) | -0.05 |  |
| Public, private & informal | -0.0002 | (0.0001) | -1.08 |  | 0.0000* | (0.0000) | -0.04 |  | 0.0000 | (0.0004) | -0.04 |  |
|  |  |  |  |  |  |  |  |  |  |  |  |  |
| Hospitalization of household members | -0.0025** | (0.0005) | -13.10 | **-13.10** | -0.0004** | (0.0001) | -0.80 | **-0.80** | 0.0003 | (0.0065) | 0.53 | **-0.80** |
|  |  |  |  |  |  |  |  |  |  |  |  |  |
|  |  |  |  |  |  |  |  |  |  |  |  |  |
| Characteristics | Panel C: Detailed decomposition:Difference due to coefficients | | | | | | | | | | | |
|  | 2005 | | | | 2010 | | | | 2016 | | | |
|  | Coefficient | Std. Err. | Percent | | Coefficient | Std. Err. | Percent | | Coefficient | Std. Err. | Percent | |
|  |  |  |  |  |  |  |  |  |  |  |  |  |
| Consumption expenditure quintile |  |  |  | **-0.25** |  |  |  | **5.17** |  |  |  | **7.62** |
| Lowest | -0.0001 | (0.0025) | -0.54 |  | -0.0015 | (0.0016) | -3.04 |  | -0.0024 | (0.0012) | -4.36 |  |
| 2nd | 0.0014 | (0.0028) | 7.34 |  | 0.0024 | (0.0024) | 4.70 |  | -0.0010 | (0.0018) | -1.83 |  |
| 3rd | -0.0010 | (0.0036) | -5.32 |  | 0.0010 | (0.0027) | 1.95 |  | 0.0078** | (0.0024) | 14.06 |  |
| 4th | -0.0030 | (0.0056) | -15.94 |  | -0.0050 | (0.0031) | -9.99 |  | -0.0023 | (0.0032) | -4.11 |  |
| Highest | 0.0027 | (0.0085) | 14.21 |  | 0.0058 | (0.0052) | 11.55 |  | 0.0022 | (0.0045) | 3.86 |  |
|  |  |  |  |  |  |  |  |  |  |  |  |  |
| Female household head | -0.0021 | (0.0036) | -11.11 | **-11.11** | -0.0034 | (0.0023) | -6.71 | **-6.71** | -0.0005 | (0.0022) | -0.91 | **-0.91** |
|  |  |  |  |  |  |  |  |  |  |  |  |  |
| Education of household head |  |  |  | **-0.35** |  |  |  | **-3.31** |  |  |  | **2.31** |
| No education | -0.0019 | (0.0059) | -9.90 |  | -0.0005 | (0.0041) | -0.96 |  | -0.0014 | (0.0034) | -2.57 |  |
| Below secondary | 0.0009 | (0.0058) | 4.85 |  | -0.0059 | (0.0040) | -11.79 |  | 0.0038 | (0.0043) | 6.78 |  |
| Secondary or above | 0.0009 | (0.0065) | 4.70 |  | 0.0047 | (0.0038) | 9.44 |  | -0.0011 | (0.0037) | -1.90 |  |
|  |  |  |  |  |  |  |  |  |  |  |  |  |
| Household size |  |  |  | **-68.29** |  |  |  | **2.70** |  |  |  | **10.71** |
| 1-2 members | 0.0009 | (0.0016) | 4.93 |  | -0.0003 | (0.0012) | -0.53 |  | -0.0010 | (0.0014) | -1.84 |  |
| 3-4 members | 0.0098 | (0.0105) | 51.72 |  | 0.0017 | (0.0053) | 3.43 |  | 0.0103* | (0.0051) | 18.47 |  |
| 5 or more members | -0.0238 | (0.0128) | -124.94 |  | -0.0001 | (0.0054) | -0.20 |  | -0.0033 | (0.0042) | -5.92 |  |
|  |  |  |  |  |  |  |  |  |  |  |  |  |
| Number of earners | -0.0012 | (0.0217) | -6.29 | **-6.29** | -0.0176 | (0.0135) | -35.03 | **-35.03** | 0.0071 | (0.0139) | 12.82 | **12.82** |
|  |  |  |  |  |  |  |  |  |  |  |  |  |
| Presence of elderly household member(s) | 0.0054 | (0.0067) | 28.41 | **28.41** | 0.0031 | (0.0039) | 6.26 | **6.26** | -0.0018 | (0.0035) | -3.16 | **-3.16** |
|  |  |  |  |  |  |  |  |  |  |  |  |  |
| Presence of children under five years | 0.0264* | (0.0123) | 138.80 | **138.80** | 0.0123 | (0.0070) | 24.61 | **24.61** | 0.0040 | (0.0063) | 7.12 | **7.12** |
|  |  |  |  |  |  |  |  |  |  |  |  |  |
| Presence of household member(s) with chronic illness | 0.0290* | (0.0124) | 152.40 | **152.40** | 0.0021 | (0.0082) | 4.23 | **4.23** | -0.0015 | (0.0081) | -2.68 | **-2.68** |
|  |  |  |  |  |  |  |  |  |  |  |  |  |
| Source of healthcare |  |  |  | **-115.24** |  |  |  | **-24.86** |  |  |  | **3.28** |
| Public only | -0.0040 | (0.0033) | -20.77 |  | -0.0039 | (0.0026) | -7.71 |  | -0.0004 | (0.0019) | -0.79 |  |
| Private only | -0.0180 | (0.0105) | -94.40 |  | -0.0144* | (0.0064) | -28.71 |  | 0.0044 | (0.0039) | 7.87 |  |
| Informal only | -0.0048 | (0.0121) | -25.03 |  | 0.0059 | (0.0069) | 11.67 |  | -0.0004 | (0.0059) | -0.76 |  |
| Public & private | -0.0013 | (0.0011) | -6.71 |  | 0.0004 | (0.0008) | 0.78 |  | 0.0002 | (0.0009) | 0.27 |  |
| Public & informal | 0.0010 | (0.0009) | 5.41 |  | 0.0011 | (0.0007) | 2.10 |  | 0.0005 | (0.0009) | 0.88 |  |
| Private & informal | 0.0048 | (0.0032) | 25.44 |  | -0.0015 | (0.0011) | -3.01 |  | -0.0024 | (0.0016) | -4.32 |  |
| Public, private & informal | 0.0002 | (0.0005) | 0.82 |  | 0.0000 | (0.0002) | 0.02 |  | 0.0001 | (0.0002) | 0.13 |  |
|  |  |  |  |  |  |  |  |  |  |  |  |  |
| Hospitalization of household members | -0.0006 | (0.0019) | -3.03 | **-3.03** | 0.0003 | (0.0011) | 0.56 | **0.56** | -0.0019 | (0.0020) | -3.34 | **-3.34** |
|  |  |  |  |  |  |  |  |  |  |  |  |  |
| Constant | 0.0129 | (0.0361) | 67.76 | **67.76** | 0.0674** | (0.0263) | 134.35 | **134.35** | 0.0368 | (0.0223) | 65.99 | **65.99** |
|  |  |  |  |  |  |  |  |  |  |  |  |  |

Std. Err. = standard error; * *p* ≤ 0.05, ** *p* ≤ 0.01
