# Supplementary material for: Rural‒urban disparities in household catastrophic health expenditure in Bangladesh: a multivariate decomposition analysis
Source: Int J Equity Health. 2024 Feb 27;23:43. doi: 10.1186/s12939-024-02125-3 (PMC10898052; doi:10.1186/s12939-024-02125-3)
Supplement: Supplementary file 7 — Additional file 7. Logit model of CHE incidence (normative food, rent, and utilities method, 40% threshold) among urban and rural households, adjusted for all covariates. [file 12939_2024_2125_MOESM7_ESM.docx]

**Additional Table 7:** Logit model of catastrophic health expenditure incidence among urban and rural households, adjusted for all covariates

| Characteristics | 2005 | | | | 2010 | | | | 2016 | | | |
| --- | --- | --- | --- | --- | --- | --- | --- | --- | --- | --- | --- | --- |
|  | Rural  (n = 2,426) | | Urban  (n = 1,373) | | Rural  (n = 4,266) | | Urban  (n = 1,967) | | Rural  (n = 15,645) | | Urban  (n = 6,371) | |
|  | Coefficient | Std. Err. | Coefficient | Std. Err. | Coefficient | Std. Err. | Coefficient | Std. Err. | Coefficient | Std. Err. | Coefficient | Std. Err. |
|  |  |  |  |  |  |  |  |  |  |  |  |  |
| Consumption expenditure quintile (ref: Lowest) |  |  |  |  |  |  |  |  |  |  |  |  |
| 2nd | -2.63** | (0.17) | -3.46** | (0.39) | -2.81** | (0.14) | -3.06** | (0.24) | -2.27** | (0.08) | -2.48** | (0.20) |
| 3rd | -3.38** | (0.20) | -4.03** | (0.42) | -3.36** | (0.15) | -3.94** | (0.38) | -2.70** | (0.09) | -3.54** | (0.25) |
| 4th | -3.59** | (0.22) | -4.89** | (0.44) | -3.63** | (0.16) | -3.49** | (0.31) | -3.04** | (0.10) | -3.38** | (0.20) |
| Highest | -3.56** | (0.23) | -4.40** | (0.43) | -3.23** | (0.17) | -3.92** | (0.31) | -3.04** | (0.11) | -3.40** | (0.23) |
|  |  |  |  |  |  |  |  |  |  |  |  |  |
| Female household head (ref: Male) | 0.12 | (0.20) | 0.30 | (0.43) | 0.10 | (0.14) | 0.32 | (0.32) | -0.15 | (0.09) | -0.31 | (0.17) |
|  |  |  |  |  |  |  |  |  |  |  |  |  |
| Education of household head (ref: No education) |  |  |  |  |  |  |  |  |  |  |  |  |
| Below secondary | -0.34* | (0.15) | -0.31 | (0.29) | -0.32** | (0.11) | -0.29 | (0.24) | -0.20** | (0.06) | -0.23* | (0.11) |
| Secondary or above | -0.89** | (0.28) | -0.89* | (0.35) | -0.68** | (0.19) | -1.20** | (0.35) | -0.67** | (0.10) | -0.68** | (0.18) |
|  |  |  |  |  |  |  |  |  |  |  |  |  |
| Household size (ref: 1-2 members) |  |  |  |  |  |  |  |  |  |  |  |  |
| 3-4 members | -0.79** | (0.23) | -0.29 | (0.69) | -0.69** | (0.16) | -0.97 | (0.31) | -0.71** | (0.08) | -0.88** | (0.24) |
| 5 or more members | -1.34** | (0.25) | -0.37 | (0.71) | -0.85** | (0.17) | -0.92** | (0.33) | -1.14** | (0.09) | -0.81** | (0.25) |
|  |  |  |  |  |  |  |  |  |  |  |  |  |
| Number of earners | -0.07 | (0.09) | -0.03 | (0.14) | -0.22** | (0.06) | -0.09 | (0.14) | -0.11* | (0.04) | -0.19* | (0.09) |
|  |  |  |  |  |  |  |  |  |  |  |  |  |
| Presence of elderly household member(s) (ref: No) | -0.09 | (0.14) | -0.27 | (0.29) | -0.04 | (0.09) | -0.32 | (0.21) | 0.19** | (0.06) | 0.28 | (0.16) |
|  |  |  |  |  |  |  |  |  |  |  |  |  |
| Presence of children under five years (ref: No) | 0.34* | (0.13) | -0.27 | (0.24) | 0.24* | (0.10) | -0.04 | (0.23) | 0.12* | (0.05) | 0.04 | (0.14) |
|  |  |  |  |  |  |  |  |  |  |  |  |  |
| Presence of household member(s) with chronic illness (ref: No) | 0.09 | (0.13) | -0.34 | (0.24) | 0.21* | (0.10) | -0.09 | (0.22) | 0.81** | (0.06) | 0.74** | (0.16) |
|  |  |  |  |  |  |  |  |  |  |  |  |  |
| Source of healthcare (ref: Public only) |  |  |  |  |  |  |  |  |  |  |  |  |
| Private only | -0.02 | (0.26) | -0.20 | (0.39) | 0.19 | (0.18) | 0.78* | (0.36) | 0.53** | (0.12) | 0.45** | (0.17) |
| Informal only | -1.09** | (0.27) | -1.95** | (0.42) | -0.67** | (0.18) | -0.65 | (0.35) | -0.69** | (0.10) | -0.61** | (0.18) |
| Public & private | 1.07 | (0.62) | 0.34 | (0.55) | 0.75** | (0.25) | 0.61 | (0.61) | 1.01** | (0.16) | 1.00** | (0.37) |
| Public & informal | 0.65 | (0.39) | -0.70 | (0.66) | 0.48 | (0.36) | -1.25 | (0.91) | 0.39* | (0.19) | 0.09 | (0.30) |
| Private & informal | 0.69* | (0.31) | -0.94* | (0.47) | 0.48 | (0.25) | 1.04 | (0.45) | 0.82** | (0.13) | 1.35** | (0.33) |
| Public, private & informal | 1.85* | (0.75) | 0.14 | (1.02) | 1.42** | (0.61) | 1.38 | (0.67) | 1.05** | (0.29) | 1.16** | (0.36) |
|  |  |  |  |  |  |  |  |  |  |  |  |  |
| Hospitalization of household members (ref: No) | 1.70** | (0.25) | 1.80** | (0.43) | 1.88** | (0.19) | 2.22** | (0.37) | 1.18** | (0.10) | 1.34** | (0.17) |
|  |  |  |  |  |  |  |  |  |  |  |  |  |
| Constant | 2.67** | (0.38) | 3.61** | (0.68) | 2.23** | (0.26) | 1.95** | (0.52) | 1.53** | (0.15) | 1.52** | (0.34) |

Note: CHE incidence is measured using the normative food, rent, and utilities method (applying WHO household equivalence scale), 40% threshold; Std. Err = standard error; * *p* ≤ 0.05, ** *p* ≤ 0.01

Note: Logit regression outputs in Stata with the "svy:" prefix do not provide a pseudo-R-squared measure. In a separate analysis (not presented here), we utilized analytic weights rather than survey commands to assess the overall goodness of fit for the identical regression model. The resulting pseudo-R-squared values for rural households were 33.01%, 31.23%, and 2303% in 2005, 2010, and 2016, respectively. The corresponding values for urban households during the same years were 32.22%, 32.18%, and 24.09%.
